# Structural modification of the tripeptide KPV by reductive “glycoalkylation” of the lysine residue

**DOI:** 10.1371/journal.pone.0199686

**Published:** 2018-06-28

**Authors:** Abigael C. Songok, Pradip Panta, William T. Doerrler, Megan A. Macnaughtan, Carol M. Taylor

**Affiliations:** 1 Department of Chemistry, Louisiana State University, Baton Rouge, Louisiana, United States of America; 2 Department of Biological Sciences, Louisiana State University, Baton Rouge, Louisiana, United States of America; Weizmann Institute of Science, ISRAEL

## Abstract

Peptides that exhibit enzymatic or hormonal activities are regulatory factors and desirable therapeutic drugs because of their high target specificity and minimal side effects. Unfortunately, these drugs are susceptible to enzymatic degradation, leading to their rapid elimination and thereby demanding frequent dosage. Structurally modified forms of some peptide drugs have shown enhanced pharmacokinetics, improving their oral bioavailability. Here, we discuss a novel glycomimetic approach to modify lysine residues in peptides. In a model system, the ε-amine of Ts-Lys-OMe was reductively alkylated with a glucose derivative to afford a dihydroxylated piperidine in place of the amine. A similar modification was applied to H-KPV-NH_2_, a tripeptide derived from the α-melanocyte stimulating hormone (α-MSH) reported to have antimicrobial and anti-inflammatory properties. Antimicrobial assays, under a variety of conditions, showed no activity for Ac-KPV-NH_2_ or the α- or ε-glycoalkylated analogs. Glycoalkylated peptides did, however, show stability toward proteolytic enzymes.

## Introduction

In the recent past, there has been a significant increase in the market for therapeutic peptides and proteins [1]. This interest is attributed to peptides’ high selectivity for their target, often with minimal side effects and toxicity [2]. Some problems that must be overcome for therapeutic peptides and proteins include proteolytic instability, immunogenicity, low oral bioavailability, and short half-life [3,4]. In order to enhance the pharmacokinetic properties of peptide drugs, various structural modifications have been effected. Examples of these modifications include *N*-methylation and the formation of cyclic peptides, which enhance membrane permeability and decrease susceptibility to enzymatic degradation [2,5]. Another strategy is to synthesize peptide analogs incorporating unnatural D-amino acids since they are less susceptible to proteolysis [6]. The half-life of a peptide can be increased using polymer conjugates, such as polyethyleneglycol (PEG) modified peptides. These PEGylated peptides have a larger hydrodynamic volume than their unmodified counterparts, which minimizes the elimination rate of the drug through renal filtration [7]. Functional mimics utilizing non-peptidic foldamers (*N*,*Nˈ*-linked oligoureas coupled to amino acid sidechains) tested positive against *S*. *aureus* [8]. Other modifications include peptide lipidation [9], hydrophobic ion pairing [10], and complexation with cyclodextrin [1]. Regardless of the nature of the modification, the multi-faceted goal is to improve the target specificity, membrane permeation, stability, solubility, and oral bioavailability of the drug without altering the therapeutic activity.

Herein we present a novel glycomimetic approach to modify the α- or ε-amino groups of lysine residues. As a model system, *Nα*-*p*-tosyl-L-lysine methyl ester (Ts-Lys-OMe) was modified at the ε-position. Having established the best chemical reaction conditions for modification, the same approach was applied to the lysine residue in the *C*-amidated tripeptide, H-KPV-NH_2_. This sequence is the carboxy-terminal tripeptide of α-melanocyte stimulating hormone, (α–MSH, Ac-SYSMEHFRWGKPV-NH_2_) [11]. Both α-MSH and Ac-KPV-NH_2_ have anti-inflammatory [11] and antimicrobial activities [11,12]. Ac-KPV-NH_2_ is more attractive for drug development compared to full-length α-MSH because α-MSH has additional activity as a melanotropic peptide [11]. In addition, Ac-KPV-NH_2_ is chemically stable and is less costly to produce because of its small size. The mechanism of the Ac-KPV-NH_2_ tripeptide’s anti-inflammatory action has received more attention than its antimicrobial activity. Elliott *et al*. reported a calcium signaling pathway for α-MSH and Ac-KPV-NH_2_, through the MC-R1 receptor [13]. They observed an elevation of intracellular calcium in human keratinocyte cells by adrenocorticotropic hormone (ACTH), α-MSH, Ac-KPV-NH_2_, and Ac-KPdV-NH_2_ (dV indicates D-valine in place of L-valine) in the presence of an adenosine agonist, which inhibits cAMP elevation [13]. Antimicrobial activity of Ac-KPV-NH_2_ has been reported for the multi-resistant human pathogens, *S*. *aureus* and *Candida albicans* [12], and antiviral activity, *viz*. HIV-1 [14]. The molecular basis for these activities remains unknown. Ac-KPV-NH_2_, along with its analogs and stereoisomers (Ac-dKPV-NH_2_, Ac-KPdV-NH_2_, Ac-KdPV-NH_2_, and Ac-dKPdV-NH_2_) [15], have similar anti-inflammatory activities to α-MSH. There are, however, conflicting studies as to whether the L-configuration of proline is essential for activity [16–18].

Antimicrobial peptides act via membrane disruption, initiated by electrostatic interactions and hydrogen bonding. Preferential affinity of such peptides for microbial membranes (typically negatively charged) rather than mammalian membranes (neutral) is attributed to their cationic nature. Giuliani *et al*. reviewed proposed disruption mechanisms in detail, including the barrel-stave, toroidal, aggregate channel, and carpet mechanisms [3]. Charnley *et al*. found that the cationic lysine residue in Ac-KPdV-NH_2_ is not essential for antimicrobial activity [19]. Replacement of lysine with alanine in the sequence (Ac-APdV-NH_2_) did not affect the activity such that a general Ac-XPdV-NH_2_ or Ac-XPV-NH_2_ sequence was proposed. Modification of H-KPV-NH_2_ at the lysine residue is therefore not expected to interfere with the antimicrobial activity of the molecule and can be used to enhance the peptide’s solubility and amphipathic properties.

**Fig 1:**
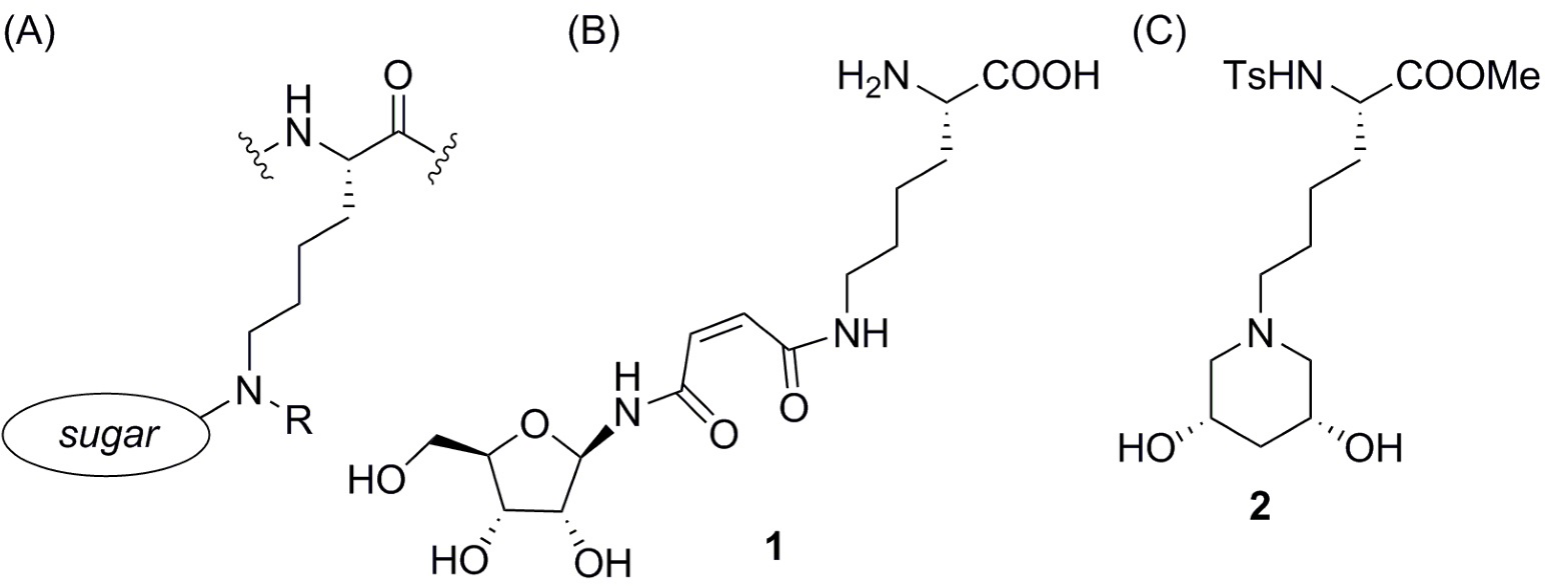
Glycoalkylated lysine. (a) Lysine modification at the ε-amine with a generic sugar molecule; (b) Glycoalkylated lysine, as described by Schlimme *et al*. [20]; (c) ε-Glycoalkylated Ts-Lys-OMe, as described in this article.

Herein we redefine the use of the term “glycoalkylation,” illustrated generically in Fig 1A as the modification of the ε-amino group of lysine. Schlimme *et al*. introduced the term for *N*-glycosylation of mono- and bicyclic dicarbonic acid imides using ribose [20]. The glycosylated imide [*e*.*g*., compound **1**, Fig 1B] was in turn used as a glycoalkylating agent for lysine through a ring-opening reaction. In this paper, the α- or ε-amino group of a lysine residue is glycoalkylated with a derivative of α-D-glucofuranose to incorporate the nitrogen into a piperidine diol ring, *e*.*g*., compound **2** in Fig 1C.

## Materials and methods

Reagents were obtained from commercial sources and used without further purification except for triethylamine, piperidine, and collidine, which were distilled after overnight stirring in CaH_2._ Methanol was dried and distilled from magnesium turnings. Silica gel for flash column chromatography was obtained from Sigma (particle size 40–63 µm). Glass TLC plates were coated with silica gel 60G F_254_ manufactured by Merck Millipore. HPLC purification was performed on a Sorbent Purity C18 300Å 5 μm column (250 × 10.0 mm) with a flow rate of 1.0 mL/min and a gradient of 20–90% acetonitrile (+ 0.1% formic acid) over 20 min, monitoring UV-absorbance 218 and 254 nm. ^1^H and ^13^C NMR spectra were recorded on a Bruker AVIII-400-Nanobay spectrometer, AV500-Prodigy or Bruker AVIII-400-3. Chemical shifts are expressed in ppm downfield of TMS, in deuterated solvents, as specified. Optical rotations were measured on a JASCO-P2000 polarimeter. High resolution mass spectrometry (HRMS) was carried out using an ESI TOF 6210 (electrospray ionization time-of-flight) mass spectrometer (Agilent Technologies). *Streptomyces griseus* pronase was purchased from VWR, and specified to be ≥45,000 proteolytic units/g dry weight. A stock solution was prepared by dissolving 2 mg of the lyophilized powder in D_2_O (2 mL).

### Chemical synthesis

#### *Nα-*Tosyl*-Nε-*1,2*-O-*isopropylidene*-α-D-*glucofuranose*-L-*lysine methyl ester (6)

Flame-dried, 4 Å molecular sieves (143.0 mg) were added to a solution of 1,2*-O-*isopropylidene*-α-D-*glucofuranose-5-carbaldehyde **4** [21, 22] (132.9 mg, 0.77 mmol, 1.0 equiv) and triethylamine (300 μL, 216.6 mg, 2.15 mmol, 2.8 equiv) in dry methanol (20 mL). Nα-*p*-Tosyl-L-lysine methyl ester hydrochloride **5** (270.9 mg, 0.77 mmol, 1.0 equiv) was added as a solid in a single portion. The mixture was stirred at rt under N_2_ for 18 h. The molecular sieves were removed by filtration, washing well with methanol. The filtrate was concentrated to give imine. ^1^H NMR (400 MHz, CDCl_3_) δ 7.59 -N = C**H**-, d, *J* = 4.5 Hz.

Sodium borohydride (40.0 mg, 1.06 mmol, 1.0 equiv) was added to a stirred solution of imine in dry methanol (15 mL) at 0°C and stirred under N_2_ for 4 h. The reaction was quenched by dropwise addition of 2M HCl (600 µL), the mixture concentrated, and the residue partitioned between EtOAc (40 mL) and water (10 mL). The aqueous layer was further extracted with EtOAc (2 x 20 mL) and the combined organic extracts were concentrated. The residue was purified by flash chromatography on silica gel, eluting with 95:5 CH_2_Cl_2_-MeOH to afford **6** as a brownish solid (240.4 mg, 66%). *R*_*f*_ 0.56 (9:1 CH_2_Cl_2_-MeOH). [α]_D_^26^ +9.53 (*c* 2.5, CHCl_3_). ^1^H NMR (400 MHz, CDCl_3_) δ 1.31 (s, 3H), 1.47–1.32 (m, 4H), 1.49 (s, 3H), 1.76–1.57 (m, 3H), 2.07 (dd, *J* = 13.2, 4.3 Hz, 1H), 2.40 (s, 3H), 2.62 (t, *J* = 6.6 Hz, 2H), 2.71 (dd, *J* = 12.4, 7.0 Hz, 1H), 2.87 (dd, *J* = 12.4, 3.3 Hz, 1H), 3.47 (s, 3H), 3.89 (dd, *J* = 7.3, 5.2 Hz, 1H), 4.37 (ddd, *J* = 14.2, 3.8, 3.5 Hz, 1H), 4.72 (t, *J* = 4.2 Hz, 1H), 5.80 (d, *J* = 3.7 Hz, 1H), 7.28 (d, *J* = 8.0 Hz, 2H), 7.70 (d, *J* = 8.3 Hz, 2H); ^13^C NMR (100 MHz, CDCl_3_) δ 21.5, 22.5, 26.1, 26.7, 28.3, 32.8, 36.5, 49.2, 52.2, 52.4, 55.6, 76.5, 80.4, 105.5, 111.0, 127.2(2C), 129.5(2C), 136.7, 143.6, 172.1. HRMS (ESI) calcd for C_22_H_35_N_2_O_7_S (M+H)^+^ 471.2159, obsd 471.2149.

#### *Nα-*Tosyl*-Nε-(*2*S*,4*R)-*dihydroxypiperidine*-L-*lysine methyl ester (2)

A solution of *Nα-*tosyl*-Nε-*1,2*-O-*isopropylidene*-α-D-*glucofuranose*-L-*lysine methyl ester (**6**) (105.0 mg, 0.24 mmol, 1.0 equiv) in TFA-water (2:1 v/v) solution was stirred for 3 h at rt. The TFA was co-evaporated with toluene, and the residue was diluted with water and lyophilized. The dried sample was dissolved in dry MeOH (3 mL) and cooled to 0°C. Sodium borohydride (30.6 mg, 0.49 mmol, 2.0 equiv) was added and stirring continued for 4 h under N_2_. The reaction was quenched by the dropwise addition of 2M HCl (0.5 mL). The mixture was concentrated, and the residue purified by flash column chromatography on silica gel eluting with 9:1 CH_2_Cl_2_-MeOH. A solution of the purified product **2** in MeOH (1 mL) was kept at 4°C, which led to crystallization (42.0 mg, 42%). *R*_*f*_ 0.37 (9:1 CH_2_Cl_2_-MeOH). [α]_D_^25^ +7.3 (*c* 1.1, CHCl_3_). ^1^H NMR (400 MHz, CDCl_3_) δ 1.21 (dt, *J* = 11.0 Hz, 1H), 1.28–1.41 (m, 2H), 1.41–1.55 (m, 2H), 1.55–1.75 (m, 2H), 1.81 (t, *J* = 9.7 Hz, 2H), 2.19–2.30 (m, 1H), 2.38 (t, *J* = 7.3 Hz, 2H) 2.45 (s, 3H), 2.94 (dd, *J* = 10.5, 3.4 Hz, 2H), 3.44 (s, 3H), 3.66–3.71 (m, 2H), 3.86 (dd, *J* = 8.6, 5.5 Hz, 1H), 7.38 (d, *J* = 8.1 Hz, 2H), 7.72 (d, *J* = 8.2 Hz, 2H); ^13^C NMR (100 MHz, CDCl_3_) δ 20.1, 22.9, 25.0, 32.1, 41.3, 51.1, 55.6, 57.4, 59.4, 59.5, 64.9(2C), 126.8 (2C), 129.2(2C), 137.8, 143.4, 172.2. HRMS (ESI) calcd for C_19_H_31_N_2_O_6_S (M+H)^+^ 415.1903, obsd 415.1904.

#### Fmoc-K(Boc)-PV-NH_2_ (10a)

*N*-Hydroxysuccinimide (143.3 mg, 1.28 mmol, 1.0 equiv) and DCC (264.1 mg, 1.28 mmol, 1.0 equiv) were added to a solution of Fmoc-Lys(Boc)-OH (600.0 mg, 1.28 mmol, 1.0 equiv) in CH_2_Cl_2_ (20 mL) at 0°C. The mixture was stirred for 20 min, warmed to rt, stirred for 4 h and filtered through a plug of cotton in a Pasteur pipette. The filtrate was concentrated, placed in the freezer for 2 h, filtered a second time and the filtrate concentrated. The residue was dissolved in DMF (6 mL) and cooled in an ice bath. To the stirred mixture was added L-proline (147.4 mg, 1.28 mmol, 1.0 equiv) and diisopropylethylamine (268 µL, 199.0 mg, 1.54 mmol, 1.2 equiv). The mixture was stirred at 0°C for 10 min, warmed to rt and stirred for 14 h. Dimethylformamide was removed by a stream of air. The residue was taken up in EtOAc (100 mL) and washed with 2M HCl (80 mL). The layers were separated, and the aqueous layer was further extracted with EtOAc (3 x 20 mL). The organic fractions were combined, filtered through anhydrous MgSO_4_ and concentrated to afford the dipeptide acid that was used directly without purification *R*_*f*_ 0.32 (9:1 CH_2_Cl_2_-MeOH).

Valine amide hydrochloride (195.4 mg, 1.28 mmol, 1.0 equiv), HATU (535.5 mg, 1.41 mmol, 1.1 equiv), and 2,4,6-collidine (340 µL, 312.8 mg, 2.58 mmol, 2.0 equiv) were added to a stirred solution of Boc-Lys(Fmoc)-Pro-OH in CH_2_Cl_2_ (6 mL) at 0°C. After 10 min, the reaction was warmed to rt and stirred for 18 h under N_2_. The mixture was concentrated and the tripeptide **10a** was isolated by flash column chromatography, eluting with 20:1 CH_2_Cl_2_-MeOH, as a colorless solid (134 mg, 44%) *R*_*f*_ 0.55 (9:1 CH_2_Cl_2_-MeOH). [α]_D_^25^ +56.7 (*c* 1.4, DMSO). ^1^H NMR (400 MHz, CD_3_OD) δ 0.94 (d, *J* = 6.1 Hz, 3H), 0.96 (d, *J* = 6.6 Hz, 3H), 1.42 (s, 9H), 1.32–1.58 (m, 4H), 1.58–1.70 (m, 1H), 1.71–1.76 (m, 1H), 2.01–2.11 (m, 5H), 2.93–3.18 (m, 2H), 3.57–3.76 (m, 1H), 3.76–3.82 (m, 1H), 4.17–4.22 (m, 2H), 4.29–4.45 (m, 3H), 4.50 (dd, *J* = 7.6, 3.9 Hz, 1H), 6.70 (d, *J* = 7.5 Hz, NH*), 7.07 (t, *J* = 5.5 Hz, NH*) 7.30 (t, *J* = 7.4 Hz, 2H), 7.39 (t, *J* = 7.4 Hz, 2H), 7.61 (d, *J* = 7.2 Hz, 2H), 7.76 (d, *J* = 7.5 Hz, 2H); ^13^C NMR (100 MHz, DMSO-*d6*) δ 18.2, 19.8, 23.0, 25.1, 28.7, 29.0, 29.7, 31.0 (2C), 39.9, 47.1 (2C), 52.9, 57.7, 59.8, 66.1, 77.8, 120.5, 125.8, 127.5, 128.0, 141.2, 144.3, 156.0, 156.6, 171.4, 171.5, 173.3; HRMS (ESI) calcd for C_36_H_50_N_5_O_7_ (M+H)^+^ 664.3705, found 664.3688.

*Does not integrate to a full proton due to proton exchange with CD_3_OD.

#### Boc-K(Fmoc)-PV-NH_2_ (10b)

Boc-Lys(Fmoc)-OH (**9b**) (600.0 mg, 1.28 mmol) was treated, by analogy to the procedure described for the conversion of **9a** to **10a**, to afford **10b** (360.0 mg, 42%) *R*_*f*_ 0.43 (9:1 CH_2_Cl_2_-MeOH). [α]_D^25^_–55.7 (*c* 0.8, MeOH). ^1^H NMR (400 MHz, CD_3_OD) δ 0.98 (d, *J* = 2.1 Hz, 3H), 0.99 (d, *J* = 2.1 Hz, 3H), 1.43 (s, 9H), 1.43–1.64 (m, 5H), 1.72–1.81 (m, 1H), 1.95–2.17 (m, 5H), 3.13 (app. t, *J* = 6.2 Hz, 2H), 3.63 (dd, *J* = 16.0 Hz, 9.6 Hz, 1H), 3.79 (dd, *J* = 16.0 Hz, 6.8 Hz, 1H), 4.17–4.22 (m 1H), 4.21 (d, *J* = 6.6 Hz, 1H), 4.27–4.34 (m, 1H), 4.35 (d, *J* = 6.9 Hz, 2H), 4.55 (dd, *J* = 8.0 Hz, 3.8 Hz, 1H), 7.31 (t, *J* = 7.4 Hz, 2H), 7.39 (t, *J* = 7.4 Hz, 2H), 7.65 (d, *J* = 7.4 Hz, 2H), 7.79 (d, *J* = 7.4 Hz, 2H); ^13^C NMR (100 MHz, CD_3_OD) δ 17.2, 18.5, 22.5, 24.7, 27.4, 28.7, 29.1, 30.6, 30.9, 39.9, 47.1, 52.4, 58.3, 60.1, 66.2, 79.2, 119.6, 124.8, 126.8, 127.4, 141.2, 144.0, 157.4, 157.5, 172.6, 172.8, 174.7; HRMS (ESI) calcd for C_36_H_50_N_5_O_7_ (M+H)^+^ 664.3705, obsd 664.3710.

#### αG’-K(Boc)PV-NH_2_ (11a)

Piperidine (552 µL, 475.8 mg, 5.59 mmol, 5.6 equiv) was added to a solution of tripeptide **10a** (797.8 mg, 1.20 mmol, 1.0 equiv) in dry DMF (20 mL). The reaction was stirred at rt for 30 min. The solvent was evaporated by a stream of air, the residue partitioned between CH_2_Cl_2_ (20 mL) and H_2_O (10 mL), and the layers separated. The aqueous layer was further washed with CH_2_Cl_2_ (3 x 10 mL) and lyophilized to afford the free amine that was used in the next reaction without further purification (*R*_*f*_ 0.59, 6:4:1 CHCl_3_-MeOH-H_2_O).

Triethylamine (250 µL, 181.4 mg, 1.79 mmol, 3.0 equiv) and flame dried 4Å powdered molecular sieves (75.0 mg) were added to a solution of tripeptide amine (263.4 mg, 0.60 mmol, 1.0 equiv) in dry MeOH (3 mL). The mixture was stirred at rt and a solution of the aldehyde (328.3 mg, 1.91 mmol, 3.2 equiv) in dry MeOH (3 mL) was added. The mixture was left to stir at rt for 24 h. The reaction was filtered through a pad of Celite^®^ that was washed well with MeOH. The filtrate was cooled to 0°C, NaBH_4_ (73.2 mg, 1.93 mmol, 3.2 equiv) was added, and the mixture was stirred for 4 h under N_2_. The reaction was quenched by dropwise addition of 2M HCl (250 µL). The mixture was concentrated, and the residue purified by flash column chromatography, eluting with 9:1 CH_2_Cl_2_-MeOH to afford the tripeptide **11a** (194 mg, 27%) *R*_*f*_ 0.54 (9:1 CH_2_Cl_2_-MeOH). [α]_D^25^_–68.7 (*c* 1.9, CHCl_3_). ^1^H NMR (500 MHz, CD_3_OD) δ 1.00 (d, *J* = 6.6 Hz, 3H), 1.01 (d, *J* = 6.8 Hz, 3H), 1.31 (s, 3H), 1.45 (s, 9H), 1.48 (s, 3H), 1.40–1.53 (m, 3H), 1.54–1.62 (m, 2H), 1.63–1.73 (m, 2H), 1.98–2.07 (m, 3H), 2.08–2.14 (m, 2H), 2.14–2.20 (m, 1H), 2.58 (dd, *J* = 12.7, 6.6 Hz, 1H), 2.79 (dd, *J* = 12.7, 3.5 Hz, 1H) 2.89–3.12 (app. t, *J* = 5.1 Hz, 3H), 3.62–3.74 (m, 2H), 3.77–3.82 (m, 1H), 4.21 (d, *J* = 6.5 Hz, 1H), 4.25–4.30 (m, 1H), 4.59 (dd, *J* = 8.2, 4.3 Hz, 1H), 4.76 (app. t, *J* = 4.2 Hz, 1H), 5.78 (d, *J* = 3.7 Hz, 1H); ^13^C NMR (125 MHz, CD_3_OD)* δ 17.1, 18.5, 22.4, 24.6, 25.0, 25.6, 27.4, 28.7, 29.4, 30.7, 32.4, 36.1, 39.7, 47.1, 50.0, 58.3, 58.8, 59.9, 77.9, 80.3, 105.7, 110.8, 157.1, 172.8 (2C), 174.2, 174,7. HRMS (ESI) calcd for C_29_H_52_N_5_O_8_ (M+H)^+^ 598.3810, obsd 598.3809.

* Reported for the major conformation only; two species were observed that were presumed to be rotamers about the prolyl amide bond.

#### Boc-K(εG’)PV-NH_2_ (11b)

Following the same series of reactions in the conversion of **10a** to **11a** above, compound **10b** (125.0 mg, 0.19 mmol) was converted to **11b** (41 mg, 60%). *R*_*f*_ 0.78 (6:4:1 CHCl_3_-MeOH-H_2_O). [α]_D^25^_–59.3 (*c* 1.1, MeOH). ^1^H NMR (400 MHz, CD_3_OD) δ 1.00 (d, *J* = 2.1 Hz, 3H), 1.01 (d, *J* = 2.1 Hz, 3H), 1.33 (s, 3H), 1.45 (s, 9H), 1.49 (s, 3H), 1.40–1.55 (m, 1H), 1.61–1.84 (m, 5H), 1.99–2.27 (m, 6H), 2.95–3.03 (m, 3H), 3.25 (dd, *J* = 12.8, 2.4 Hz, 1H), 3.66–3.27 (m, 1H), 3.83–3.88 (m, 1H), 4.18 (d, *J* = 6.6 Hz, 1H), 4.37 (t, *J* = 6.7 Hz, 1H), 4.42–4.48 (m, 1H), 4.55 (dd, *J* = 8.2, 4.2 Hz, 1H), 4.84 (t, *J* = 4.1 Hz, 1H), 5.89 (d, *J* = 3.5 Hz, 1H); ^13^C NMR (100 MHz, CD_3_OD,) δ 17.2, 18.5, 22.3, 24.7, 25.0, 25.7, 25.9, 27.3, 29.0, 30.7, 30.8, 36.2, 47.4, 48.0, 50.7, 52.0, 58.5, 60.3, 74.0, 79.2, 80.4, 105.9, 111.3, 156.5, 172.2, 172.9, 174.7. HRMS (ESI) calcd for C_29_H_51_N_5_O_8_ (M+H)^+^ 598.3810, obsd 598.3817.

#### αG*-KPV-NH_2_ (12a)

A solution of compound **11a** (78.0 mg, 0.13 mmol, 1.0 equiv) in TFA-H_2_O (2:1 v/v, 4.5 mL) was stirred for 3.5 h. The mixture was diluted with toluene (20 mL) and concentrated. The residue was dissolved in MeOH and stirred at 0°C. Solid NaHCO_3_ (35.3 mg) was added to neutralize the solution. NaBH_3_CN (16.3 mg, 0.26 mmol, 2.0 equiv) was added and the mixture stirred for 15 h. The reaction was quenched by the dropwise addition of 2M HCl (~600 µL), concentrated, and the residue subjected to HPLC to afford compound **12a** (17.8 mg, 31%). t_R_ 15.5 min. *R*_*f*_ 0.13 (6:4:1 CHCl_3_-MeOH-H_2_O). [α]_D_^25^–28.9 (*c* 0.1, MeOH); ^1^H NMR (500 MHz, CD_3_OD) δ 1.01 (d, *J* = 6.8 Hz, 3H), 1.02 (d, *J* = 6.7 Hz, 3H), 1.24 (app. q, *J* = 10.7 Hz, 1H), 1.29–1.52 (m, 2H), 1.62–1.78 (m, 3H), 1.79–1.92 (m, 1H), 1.94–2.13 (m, 5H), 2.15–2.28 (m, 2H), 2.31 (t, *J* = 10.0 Hz, 1H), 2.87–3.02 (m, 4H), 3.56 (dd, *J* = 10.1, 3.9 Hz, 1H), 3.60–3.78 (m, 3H), 3.88–3.92 (m, 1H), 4.18 (d, *J* = 6.8 Hz, 1H), 4.53 (dd, *J* = 8.4, 4.0 Hz, 1H); ^13^C NMR (125 MHz, CD_3_OD) δ 17.2, 18.4, 22.7, 24.4, 24.5, 27.0, 29.4, 30.7, 39.2, 41.6, 47.4, 55.3, 56.9, 58.5, 60.3, 65.2, 65.7 (2C), 171.0, 173.2, 174.7; HRMS (ESI) calcd for C_21_H_40_N_5_O_5_ (M+H)^+^ 442.3024, obsd 442.3029.

#### H-K(εG*)PV-NH_2_ (12b)

By analogy to the procedure described for conversion of **11a** to **12a,** compound **11b** (139.0 mg, 0.23 mmol) was converted to **12b**. The crude product was purified by HPLC to afford ε-glycoalkylated **12b** (24 mg, 23%). t_R_ 15.6 min. *R*_*f*_ 0.20 (6:4:1 CHCl_3_-MeOH-H_2_O). [α]_D^25^_–35.5 (*c* 0.4, MeOH). ^1^H NMR (500 MHz, CD_3_OD) δ 1.00 (d, *J* = 6.8 Hz, 3H), 1.01 (d, *J* = 6.7 Hz, 3H), 1.19–1.35 (m, 1H), 1.41–1.54 (m, 2H), 1.42–1.64 (m, 2H), 1.66–1.76 (m, 1H), 1.78–1.92 (m, 3H), 1.96–2.16 (m, 4H), 2.20–2.29 (m, 2H), 2.50 (app. t, *J* = 7.7 Hz, 2H), 2.98 (dd, *J* = 10.6, 3.7 Hz, 2H), 3.63–3.66 (m, 1H), 3.68–3.75 (m, 3H), 3.91 (t, *J* = 6.2 Hz, 1H), 4.20 (d, *J* = 6.7 Hz, 1H), 4.58 (dd, *J* = 8.1, 4.6 Hz, 1H); ^13^C NMR (125 MHz, CD_3_OD) δ 17.1, 18.4, 22.5, 24.6, 25.8, 28.8, 30.7, 32.4, 41.7, 47.2, 51.9, 57.5, 58.4, 59.6 (2C), 60.1, 65.0 (2C), 171.4, 172.6, 174.7. HRMS (ESI) calcd for C_21_H_40_N_5_O_5_ (M+H)^+^ 442.3024, obsd 442.3032.

#### Ac-KPV-NH_2_ (12c)

A solution of tripeptide **10a** (362.2 mg, 0.55 mmol, 1.0 equiv) in piperidine (544 µL, 470.0 mg, 5.50 mmol, 10.0 equiv) and DMF (5 mL) was stirred for 30 min. The solvent was evaporated by a stream of air, and the residue partitioned between CH_2_Cl_2_ (50 mL) and H_2_O (30 mL). The aqueous layer was further washed with CH_2_Cl_2_ (2 x 20 mL) and lyophilized to afford the free amine (232 mg, 96%). *R*_*f*_ 0.28 (9:1 CH_2_Cl_2_-MeOH).

A portion of the free amine (94 mg, 0.213 mmol) was dissolved in a mixture of Ac_2_O-pyridine (1:1 v/v, 6 mL) and stirred for 15 h, concentrated and purified by flash column chromatography, eluting with 100:7 CH_2_Cl_2_-MeOH to give the acetylated tripeptide, Ac-K(Boc)-PV-NH_2_ (72 mg, 70%). *R*_*f*_ 0.50 (20:3 CH_2_Cl_2_-MeOH). [α]_D^25^_–65.4 (*c* 1.5, CHCl_3_). ^1^H NMR (400 MHz, CD_3_OD) δ 0.99 (d, *J* = 3.4 Hz, 3H), 1.00 (d, *J* = 3.4 Hz, 3H), 1.43–1.56 (m, 4H), 1.45 (s, 9H), 1.59–1.72 (m, 1H), 1.77–1.87 (m, 1H), 1.96–2.19 (m, 5H), 1.98 (s, 3H), 3.06 (app. t, *J* = 6.0 Hz, 2H), 3.66–3.73 (m, 1H), 3.73–3.92 (m, 1H), 4.22 (app. t, *J* = 6.3 Hz, 1H), 4.54 (dd, *J* = 8.0, 3.9 Hz, 1H), 4.54–4.59 (m, 1H), 6.59 (br s, NH*), 7.91 (d, *J* = 8.2 Hz, NH*), 8.18 (d, *J* = 7.0 Hz, NH*); ^13^C NMR (100 MHz, CD_3_OD) δ 17.1, 18.5, 20.9, 22.6, 24.7, 27.4(3C), 28.8, 29.3, 30.7 (2C), 39.6, 47.3, 51.3, 58.3, 60.1, 78.4, 157.1, 171.8, 171.9, 172.8, 174.7. HRMS (ESI) calcd for C_23_H_42_N_5_O_6_ (M+H)^+^ 484.3135, obsd 484.3130.

*Does not integrate for a full proton due to deuterium exchange.

The acetylated tripeptide, Ac-K(Boc)PV-NH_2_ (72.0 mg, 0.19 mmol) was dissolved in a mixture CH_2_Cl_2_-TFA (1:1 v/v, 4 mL) and stirred at rt for 30 min. The mixture was concentrated, and the residue dissolved in toluene and concentrated again. The residue was purified by HPLC to afford the free amine **12c** (40.8 mg, 71%). t_R_ 16.2 min. *R*_*f*_ 0.36 (20:13:3:1 CHCl_3_-MeOH-H_2_O-NH_3_). [α]_D_^25^ (*c* 0.6, MeOH). ^1^H NMR (400 MHz, CD_3_OD) δ 1.00 (d, *J* = 2.5 Hz, 3H), 1.01 (d, *J* = 2.5 Hz, 3H), 1.42–1.55 (m, 2 H), 1.62–1.73 (m, 3 H), 1.75–1.84 (m, 1 H), 1.96–2.15 (m, 4H), 1.99 (s, 3H), 2.18–2.24 (m, 1H), 2.78 (t, *J* = 7.0 Hz, 2H), 3.66–3.71 (m, 1H), 3.86–3.92 (m, 1H), 4.18 (d, *J* = 6.9 Hz, 1H), 4.53 (dd, *J* = 8.4, 4.6 Hz, 1H), 4.60 (dd, *J* = 8.1, 5.9 Hz, 1H); ^13^C NMR (100 MHz, CD_3_OD) δ 17.1, 18.5, 20.9, 22.2, 24.7, 29.0 (2C), 30.6, 30.7, 39.7, 47.4, 51.1, 58.5, 60.1, 171.6, 171.8, 172.9, 174.7; HRMS (ESI) calcd for C_18_H_34_N_5_O_4_ (M+H)^+^ 384.2611, obsd 384.2606.

#### Determination of stability of tripeptides to pronase

To a solution of each tripeptide* in D_2_O (300 µL) was added 1M NH_4_HCO_3_ (20 µL) and 50 mM CaCl_2_ (40 µL). The pH of the resulting solution was adjusted to 7.0 by the addition of 3.7% HCl (10–12 µL). The volume was adjusted to 395 µL and the ^1^H-NMR spectrum recorded at 500 MHz. An aliquot (2 µL) of the 2 mg/mL pronase stock solution was added to the solution of tripeptide and the ^1^H-NMR spectrum recorded at 15 min intervals for 1 h at RT. The solution was warmed to 37°C using the NMR spectrometer’s variable temperature controller, and spectra recorded, at 15 min intervals, for 2 h. The reaction was then incubated in an Imperial III incubator (LabLine) at 37°C and transferred briefly to the NMR probe at room temperature periodically to monitor the reaction.

*100 µg G*-KPV-NH_2_ (**12a**); 200 µg K(G*)PV-NH_2_ (**12b**) and 200 µg Ac-KPV-NH_2_ (**12c**)

## Results and discussion

### Chemical synthesis

**Fig 2:**
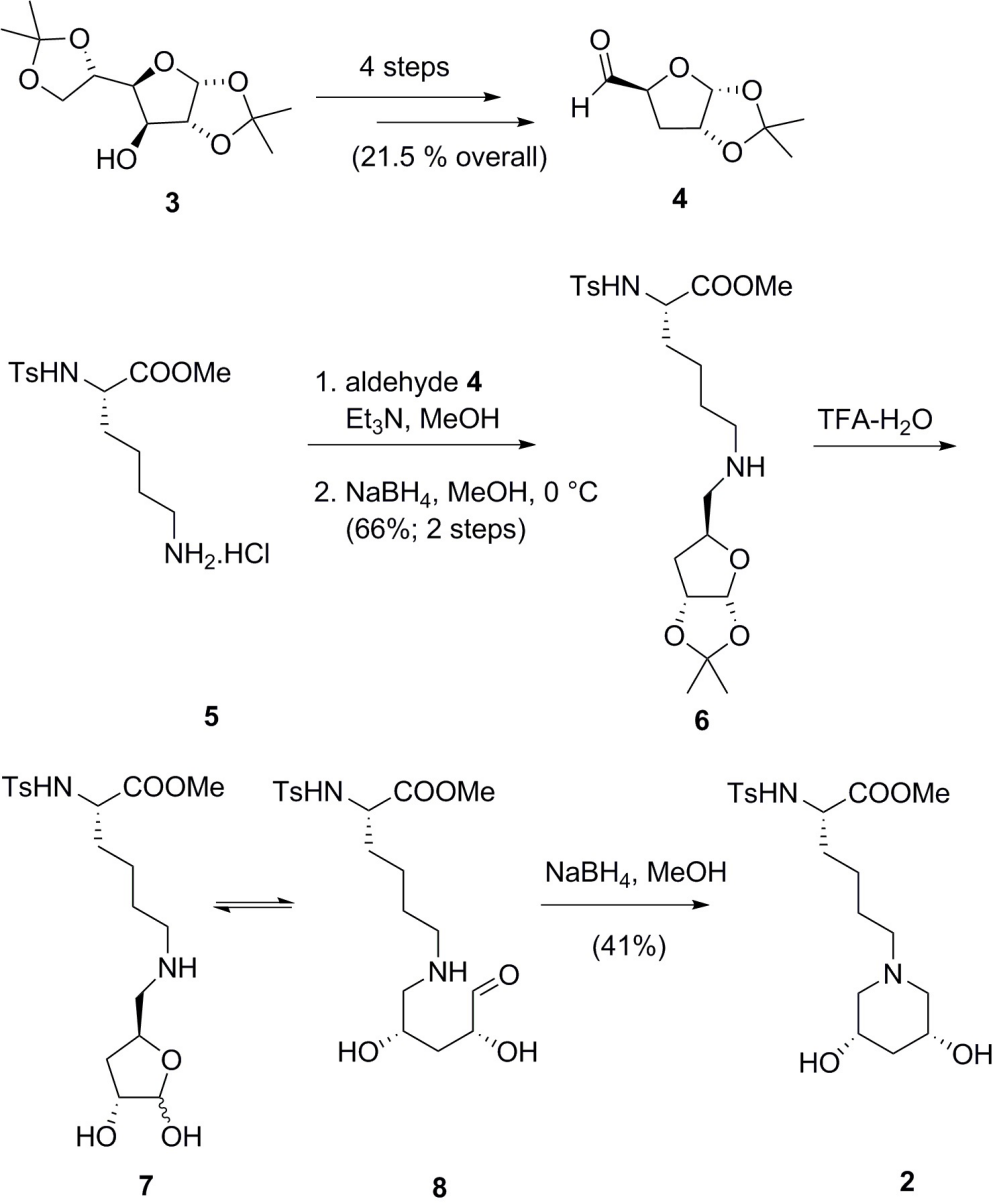
Reaction scheme 1. Synthesis of ε-glycoalkylated Ts-Lys-OMe (**2**).

Aldehyde **4** was prepared from commercially available 1,2:5,6-di-*O*-isopropylidene-α-D-glucofuranose (**3**) according to literature procedures (Fig 2) [21–27]. Specifically, Barton-McCombie deoxygenation at C-3 [23,24], selective hydrolysis of the less substituted acetal and oxidative cleavage of the 5,6-diol afforded the requisite aldehyde **4** [21,22,27]. Aldehyde **4** has been subjected to reductive amination previously with benzylamine [28]. Hydroxylated piperidines have been prepared previously by condensation of carbohydrate-derived 1,5-dialdehydes with an amine [29–31]. Indeed, Steiner *et al*. performed such a “double reductive deamination” with the ε-amino group of Boc-L-Lys-OMe en route to β-xylosidase inhibitors [32].

In the current context, we sought to perform two sequential glycoalkylations in a controlled fashion. Aldehyde **4** was condensed with the ε-amino group of lysine derivative **5**. Evidence for imine formation was afforded by ^1^H NMR: there was no residual aldehyde signal (δ 9.68 ppm, RC**H** = O, d, *J* = 1.9 Hz) and the imine gave rise to a distinct new signal (δ 7.59 ppm, RC**H** = N, d, *J* = 4.5 Hz). Following verification of imine formation, reduction was performed under standard conditions to give the secondary amine **6**.

The next step in the synthesis of the 3,5-piperidinediol involved liberation of the masked aldehyde followed by an intramolecular reductive amination. Acid hydrolysis of the remaining acetal led to an equilibrium mixture of compounds: the two anomers of hemiacetal **7** and the open chain aldehyde **8**. Reduction of the cyclic iminium ion led to formation of piperidine **2**.

**Fig 3:**
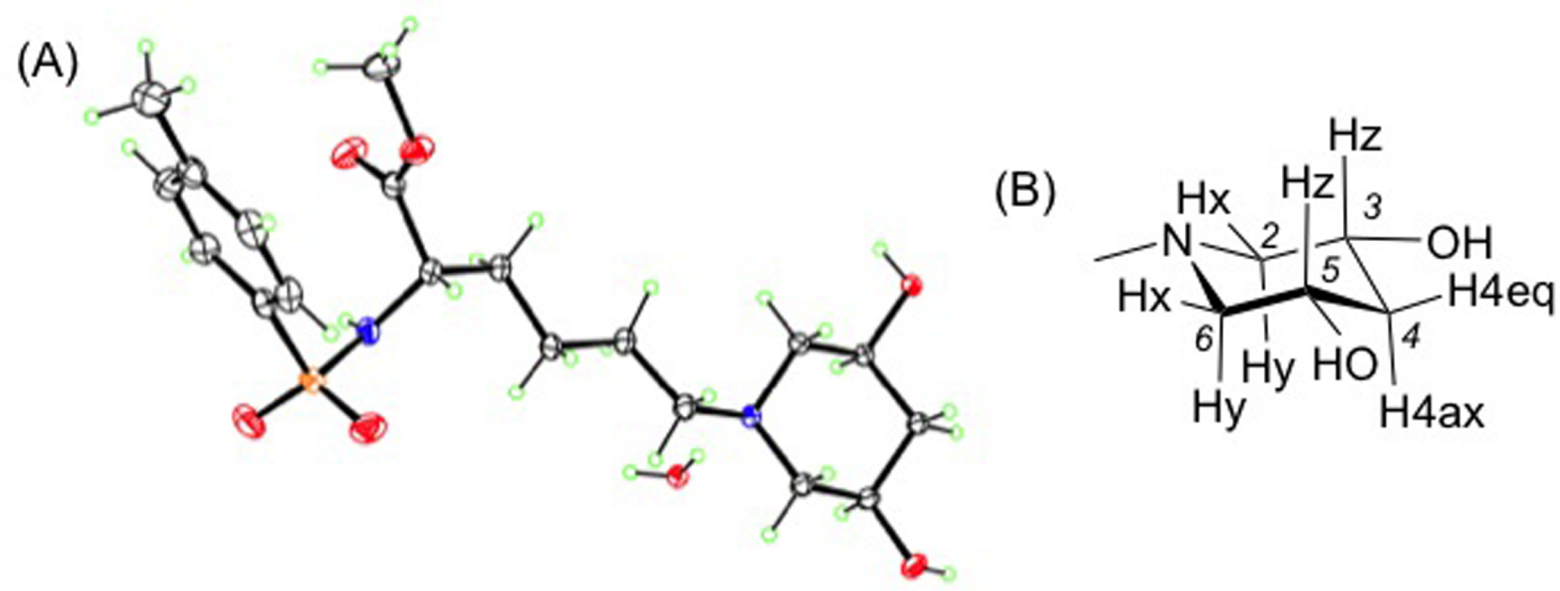
Structure of compound 2. (a) ORTEP of the hydrate of compound **2** as determined by X-ray crystallography; (b) Piperidine ring of compound **2** showing the three pairs of equivalent hydrogens Hx, Hy, and Hz.

From the crystal structure of compound **2**, shown in Fig 3A, the piperidine-2,4-diol ring is symmetric along the ring plane passing through N and C4. Each hydroxyl group of the diol adopts an equatorial orientation. ^1^H NMR analysis of compound **2** confirmed the symmetry of the piperidine, showing three pairs of equivalent protons, Fig 3B: Hx (H2e and H6e); Hy (H2a and H6a); and Hz (H3 and H5). A doublet of doublet peak was observed at δ 2.94 corresponding to H2e, H6e with a large geminal coupling constant (*J*_2e,2a_ and *J*_6e,6a_ = 10.4 Hz) and a small vicinal coupling constant (*J*_2e,3a_ and *J*_6e,5a_ = 3.3 Hz). This small vicinal coupling constant places H3 and H5 in axial positions, consistent with the equatorial orientation of the hydroxyl groups in the crystal structure.

**Fig 4:**
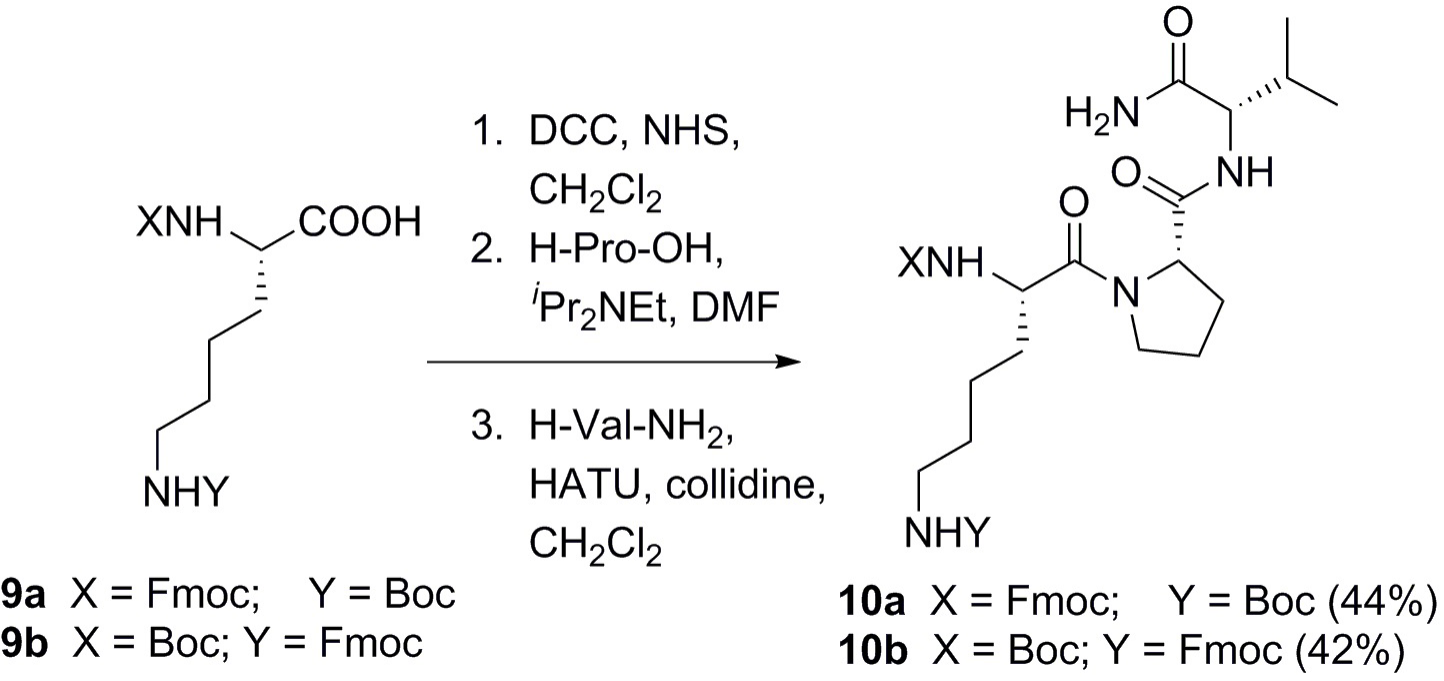
Reaction scheme 2. Synthesis of H-KPV-NH_2_ derivatives **10a** and **10b**.

Having confirmed the structure and determined reaction conditions for “glycoalkylation,” similar conditions were utilized to modify the α- or ε-amino groups of the lysine residue in the tripeptide H-KPV-NH_2_. For site-specific modification, the lysine building block in the tripeptide synthesis had orthogonal protecting groups. For α-modification, the protecting groups were Boc at the ε-position and Fmoc at the α-position. The protecting groups were switched for the ε-modification (Fig 4).

**Fig 5:**
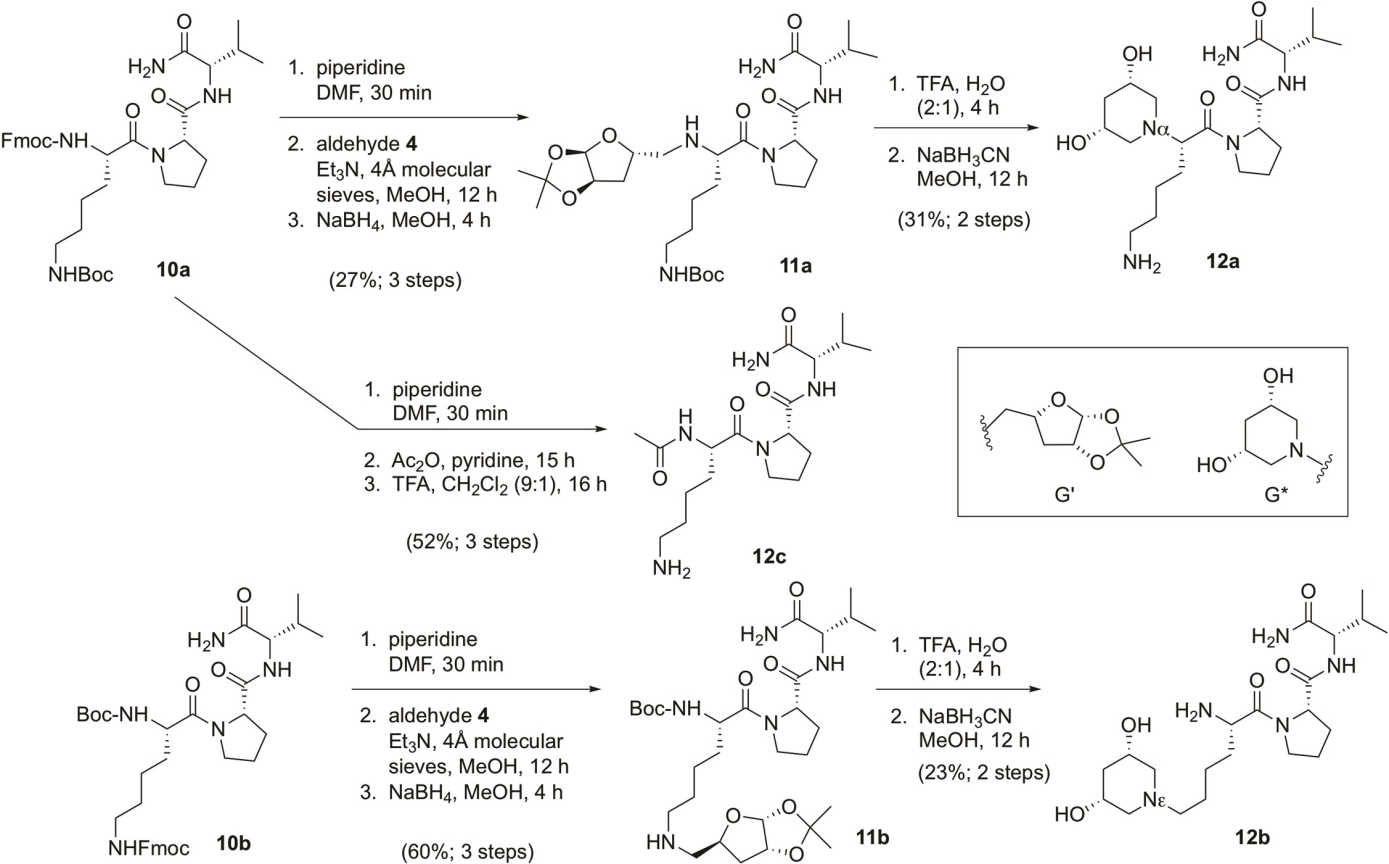
Reaction scheme 3. Synthesis of compounds **12a**, **12b**, and **12c**.

Three derivatives of H-KPV-NH_2_ were prepared to test for activity against *S*. *aureus* and stability toward proteases: αG*-KPV-NH_2_ (**12a**), H-K(εG*)PV-NH_2_ (**12b**) and Ac-KPV-NH_2_ (**12c**). The abbreviation G* represents the dihydroxylated piperidine in place of the α-NH_2_ or ε-NH_2_ group in compounds **12a** and **12b** respectively. The end-capped tripeptide Ac-KPV-NH_2_ (**12c**) was intended as a positive control. For both the α- and ε-modification, Fmoc deprotection of the tripeptide (**10a** or **10b**) led to the free amine at the α- or ε-position, respectively. Each free amine was condensed with aldehyde **4** by reductive alkylation to afford tripeptides **11a** and **11b** (Fig 5, with the sugar being designated as G’ in the furan form). The 1,2-acetonide functionality in compounds **11a** and **11b** was cleaved in TFA-water, liberating an aldehyde that underwent reductive aminocyclization to form the 3,5-dihydroxypiperidine ring at the α and ε-positions, respectively. Ac-KPV-NH_2_ (**12c**) was synthesized from compound **10a**, in order to compare the activities of the two derivatives **12a** and **12b** with the activity of **12c** as previously reported in the literature. Fmoc deprotection of **12a**, acetylation of the resulting amine with acetic anhydride, and Boc deprotection with TFA afforded **12c**.

### Biological assays

The sensitivity of various bacterial strains was tested using the agar diffusion method [33–36] with the compounds **12a-c** that we had synthesized. Details are provided in S1 File. Whilst the positive control, ampicillin, showed inhibition of bacterial growth, no inhibition zones were observed for the negative control, water, and compounds **12a-c**.

To verify the activity of Ac-KPV-NH_2_ (**12c**), the peptide was purchased from Bachem (Bubendorf, Switzerland), the same supplier as was used by Charnley *et al*. [19], following protocols similar to those reported by Cutuli *et al*. [12] and Charnley *et al*. [19]. Details are provided in S1 File. Again, no inhibition of bacterial growth was observed.

These results were surprising and disappointing because Ac-KPV-NH_2_ (**12c**) has been reported as an anti-microbial agent [12,19,37]. The original report by Catania and coworkers in 2000 described activity against both *Staphylococcus aureus* and *Candida albicans*, with effects over a broad range of concentrations, including “the physiological (picomolar) range [12].” In 2009, there was debate over the original report of antifungal activity [38,39]. Singh and Mukhopadhyay independently described the 90% staphylocidal activity of Ac-KPV-NH_2_ (**12c**) at micromolar concentrations and 50% activity in the nanomolar concentration range [37]. Charnley *et al*. reported broad range activity against both Gram-positive and Gram-negative bacteria [19]. On the other hand, without further discussion, Grieco *et al*. stated that “these molecules have weak activity in standard microbiology conditions and this hampers a realistic clinical use [40].” Lau *et al*. recently performed direct comparisons of 30 ultra-short antimicrobial peptides against *Staphylococcus aureus*, *Pseudomonas aeruginosa*, and *Candida albicans* [41]. Their study included five tripeptides, Ac-KPV-NH_2_ (**12c**) amongst them; none of the tripeptides were active against the panel of skin pathogens, indicating MICs greater than 100 μM.

While the compounds did not show any antimicrobial activity under the variety of conditions tested, the impact of glycoalkylation could be assessed vis-à-vis improved stability to proteolytic enzymes. Pronase is a commercially-available cocktail of enzymes used routinely to digest proteins to their constituent amino acids [42]. Each of the three peptides (**12a-c**) was treated with pronase, and the composition of the mixture monitored by ^1^H NMR spectroscopy (see S1 File). The “parent” peptide, Ac-KPV-NH_2_ (**12c**) was degraded to its three constituent amino acids within 24 hours. The signal attributable to Hα of the proline (P) residue shifted upfield by about 0.2 ppm, with a concomitant change from an apparent triplet (in the tripeptide) to a doublet of doublets in the free amino acid, consistent with a change in conformation of the pyrrolidine ring. The signal attributable to Hα of the valine (V) residue shifted upfield by nearly 0.5 ppm. These upfield shifts are in accordance with removal of the electron-withdrawing *N*-acyl group in each case. The α-glycoalkylated tripeptide (**12a**) was completely stable under the conditions of the pronase experiment. Less clear-cut was the behavior of the ε-glycoalkylated tripeptide (**12b**). The peptide appears to be stable, with Hα signals of both P and V remaining well-defined and with the same chemical shift and the molecular ion was still evident in the mass spectrum. The broad signals assigned to Hε and the protons of the piperidine ring reflect the dynamic nature of the Lys side chain. Upon prolonged incubation with the mixture of proteolytic enzymes, perhaps undergoing autoproteolysis, these signals generally moved upfield and became broader.

## Conclusions

We have developed the reaction chemistry to produce regioselectively glycoalkylated peptides. Specifically, reductive amination of D-glucose-derived aldehyde **4** with either the α- or ε-amino group of lysine residues gave a secondary amine. Upon liberation of the aldehyde derived from the anomeric carbon of glucose, an intramolecular reductive amination could be induced to afford a dihydroxylated piperidine moiety. Acknowledging that the impact of such a modification on biological activity is unlikely to be generalizable to peptides of assorted classes, we sought to study the effect glycoalkylation on the antibacterial activity of Ac-KPV-NH_2_ (**12c**). Unfortunately, during the course of our work, controversy arose in the literature surrounding its alleged antimicrobial activity. Like others, we were unable to reproduce the results under a number of assay conditions. Nevertheless, we have shown that the internal peptide bonds of the glycoalkylated tripeptides, **12a** and **12b**, are stable over several days to pronase. Future work will involve application of the glycoalkylation concept to other sequences and we trust that this approach will appeal to others interested in improving the bioavailability, solubility and half-life of lysine-containing peptides.

## Supporting information

S1 File Procedures and NMR spectra. Experimental procedures for the synthesis of aldehyde **4**, ^1^H and ^13^C NMR spectra for the compounds involved in the synthesis of aldehyde **4**, and ^1^H and ^13^C NMR spectra for the compounds in reaction schemes 1 (Fig 2), 2 (Fig 4), and 3 (Fig 5), computing details, atom coordinates, bond lengths and angles from the X-ray structure determination of compound **2**; NMR spectra over the timecourse of the pronase-stability experiments.
(PDF)

S2 File Crystallographic information file. Crystallographic information file for the hydrate of compound **2** as determined by X-ray crystallography. Data has been deposited at the CCDC with deposition number 1825648.
(CIF)
