# Supplementary material for: Structural modification of the tripeptide KPV by reductive “glycoalkylation” of the lysine residue
Source: PLoS One. 2018 Jun 28;13(6):e0199686. doi: 10.1371/journal.pone.0199686 (PMC6023233; doi:10.1371/journal.pone.0199686)
Supplement: S1 File — Experimental procedures for the synthesis of aldehyde 4, 1H and 13C NMR spectra for the compounds involved in the synthesis of aldehyde 4, and 1H and 13C NMR spectra for the compounds in reaction schemes 1 (Fig 2), 2 (Fig 4), and 3 (Fig 5), computing details, atom coordinates, bond lengths and angles from the X-ray structure determination of compound 2; NMR spectra over the timecourse of the pronase-stability experiments. (PDF) [file pone.0199686.s001.pdf]

Supporting Information

Structural modification of the antimicrobial tripeptide KPV by reductive "glycoalkylation" of the lysine residue

Abigael C. Songok[1], Pradip Panta[2], William T. Doerrler[2], Megan A. Macnaughtan[1], and Carol M. Taylor[1]*

[1]Department of Chemistry, Louisiana State University, Baton Rouge, Louisiana 70803, United States

[2]Department of Biological Sciences, Louisiana State University, Baton Rouge, Louisiana 70803, United States

Table of Contents

Experimental Procedures for Synthesis of Aldehyde **4** S2

^{1}H and ^{13}C NMR Spectra of Compounds in Scheme S1 .. .. S5

^{1}H and ^{13}C NMR Spectra of Compounds in Scheme 1 .. .. S13

^{1}H and ^{13}C NMR Spectra of Compounds in Scheme 2 .. .. S17

^{1}H and ^{13}C NMR Spectra of Compounds in Scheme 3 .. .. S21

Details of X-Ray Structure Determination of Compound **2** S33

Antibacterial Assays and Results S39

Stability of Peptides to Pronase as Monitored by NMR S43

Experimental Procedures for Synthesis of Aldehyde 4

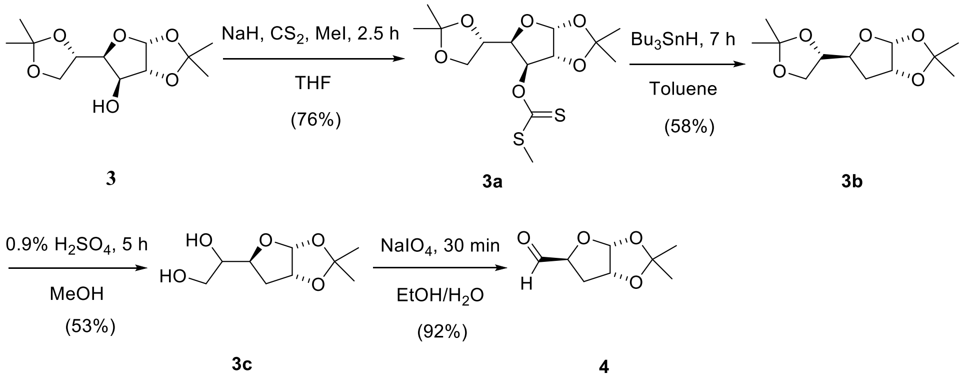

Scheme S1: Synthesis of the aldehyde **4**

1,2:5,6-Di-*O*-isopropylidene-*3-O-(*S-methyldithiocarbonate*)-α-D*-glucofuranose 3a [23,24,26]. A solution of 1,2:5,6-di-*O*-isopropylidene-*α-D*-glucofuranose (4.006 g, 15.39 mmol,1.00 equiv) and imidazole (4 mg, 0.58 mmol, 0.03 equiv) in THF (100 mL) was stirred at 0 ºC under a nitrogen atmosphere. 60% dispersion of NaH (1.26 g, 31.50 mmol, 2.05 equiv) was added in portions over 10 min.The reaction was warmed to rt and stirred for 30 min. To the reaction mixture was added CS_2 (3.00 mL, 3.78 g, 49.64 mmol 3.23 equiv) resulting in a bright yellow solution. After 45 min, MeI (1.92 mL, 4.39 g, 30.84 mmol, 2.00 equiv) was added and stirring continued for 50 min under nitrogen at rt. The reaction was quenched by addition of glacial acetic acid (≈ 2 mL), the mixture was filtered and the filtrate concentrated. The residue was taken up in ethyl acetate (150 mL), washed with $NaHCO_3$ (3 x 80 mL) and dried over anhydrous $MgSO_4$, filtered and concentrated. The residue was purified by flash column chromatography eluting with 10:1 hexanes-EtOAc to afford **3a** as a yellow crystalline solid (4.13 g, 76%). R_f 0.38 (8:1 hexanes-EtOAc). $[\alpha]_D^{25}$ = - 27.8 (*c* 2.3, $CHCl_3$). Lit.[26] $[\alpha]_D^{25}$ = - 23.6 (*c* 8.1, $CHCl_3$). ^1H NMR (400 MHz, $CDCl_3$) δ 1.25 (s, 6H), 1.34 (s, 3H), 1.46 (s, 3H), 2.53 (s, 3H), 4.03-4.12 (m, 2H), 4.27-4.19 (m, 2H), 4.67(d, *J* = 3.8 Hz, 1H), 5.85-5.83 (m, 2H); ^{13}C NMR

(100 MHz, $CDCl_3$) δ 19.3, 25.3, 26.3, 26.7, 26.8, 67.0, 72.4, 79.7, 82.8, 84.2, 105.0, 109.4, 112.4, 214.8. HRMS (ESI) calcd for $C_{14}H_{23}O_6S_2$ $(M+H)^+$ 351.0931, obsd 351.0938.

3-Deoxy-1,2:5,6-di-*O*-isopropylidene-*α-D*-glucofuranose 3b [23,24,26]. A solution of 1,2:5,6-*Di-O*-isopropylidene-*3-O-(*S-methyldithiocarbonate*)-α-D*-glucofuranose (2.81 g, 8.02 mmol, 1.0 equiv) in toluene (60 mL) was added as a whole to a solution of Bu_3SnH (5 mL, 5.4 g 18.58 mmol, 2.3 equiv) in toluene (60 mL). The mixture was stirred on reflux (128 ºC) under N_2 for 7 h. The mixture turned from bright yellow to pale yellow as the reaction progressed. The solvent was removed under low pressure, the residue dissolve in acetonitrile (200 mL) and washed with petroleum ether (3 x 65 mL). The acetonitrile layer was concentrated and the residue purified by flash column chromatography, eluting with 3:1 hexanes-EtOAc to give **3b**, as a colorless viscous liquid (1.14 g, 58%). R_f 0.28 (3:1 hexanes-EtOAc). $[\alpha]_D^{25}$ = - 7.93 (*c* 5.19, $CHCl_3$). Lit.[26] $[\alpha]_D^{22}$ = - 7.60 (*c* 1.8, $CHCl_3$). ^1H NMR (400 MHz, $CDCl_3$) δ 1.28 (s, 3H), 1.32 (s, 3H), 1.39 (s, 3H), 1.47 (s, 3H), 1.7 (ddd, *J* = 13.5, 10.0, 4.8 Hz, 1H), 2.15 (dd, *J* = 13.6, 4.0 Hz, 1H), 3.7 (ddd, *J* = 8.8, 6.7, 4.2 Hz, 1H), 4.04-4.15 (m, 3H), 4.72 (t, *J* = 4.4 Hz, 1H), 5.70 (d, *J* = 3.6 Hz, 1H); ^{13}C NMR (100 MHz, $CDCl_3$) δ 25.1, 25.6, 26.4, 26.7, 35.2, 67.1, 76.7, 78.5, 80.3, 105.5, 109.5, 111.2. HRMS (ESI) calcd for $C_{12}H_{21}O_5$ $(M+H)^+$ 245.1384, obsd 245.1387.

3-Deoxy-1,2-*O*-isopropylidene-*α-D*-glucofuranose 3c [26]. A solution of 3-deoxy-1,2:5,6-di-*O*-isopropylidene-*α-D*-glucofuranose (1.025 g, 4.19 mmol) in methanol (20 mL) was stirred at rt, followed by dropwise addition of 0.9 % H_2SO_4 (5 mL) over 5 min. The mixture was stirred for 5 h, neutralized with saturated $NaHCO_3$ (3 mL) and extracted with EtOAc (3 x 30 mL). The combined organic layers were dried over anhydrous $MgSO_4$, filtered, concentrated and purified by flash column chromatography eluting with 9:1 EtOAc-Hexanes, to afford compound **3c** as a colorless crystalline solid (450.1 mg, 53 %) R_f 0.24 (9:1 EtOAc-Hexanes). $[\alpha]_D^{25}$ = -17.2 (*c* 2.80,

$CHCl_3$). Lit.[26] $[\alpha]_D$ = -14 (*c* 1.2, EtOH) ^1H NMR (400 MHz, $CDCl_3$) δ 1.33 (s, 3H), 1.51 (s, 3H), 1.85 (ddd, *J* = 13.5, 10.7, 4.8 Hz, 1H), 2.07 (dd, *J* = 13.5, 4.5 Hz, 1H), 2.59 (t, *J* = 5.4 Hz, 1H), 2.87 (d, *J* = 3.9 Hz, 1H), 3.56-3.62 (m, 1H), 3.65-3.74 (m, 1H), 3.91 (dt, *J* = 3.7, 7.5 Hz, 1H), 4.22 (td, *J* = 10.7, 4.4 Hz, 1H) 4.76 (t, *J* = 4.2 Hz, 1H), 5.81 (d, *J* = 3.7 Hz, 1H); ^{13}C NMR (100 MHz, $CDCl_3$) δ 26.0, 26.7, 33.6, 63.5, 72.1, 78.5, 80.5, 105.1, 111.3; HRMS (ESI) calcd for $C_9H_{17}O_5$ $(M+H)^+$ 205.1071, obsd 205.1073.

1,2-*O*-Isopropylidene-*α-D*-glucofuranose-5-carbaldehyde 4 [21]. A solution of 3-Deoxy-1,2-*O*-isopropylidene-*α-D*-glucofuranose (405 mg, 1.98 mol, 1 equiv) in ethanol (2 mL) was added to a stirred solution of $NaIO_4$ (1.048 g, 4.90 mol, ~ 2.5 equiv) in water (10 mL). The reaction mixture was stirred for 30 min at rt, diluted with ethanol (60 mL), and filtered. The filtrate was evaporated, taken up in the EtOAc and filtered through anhydrous $MgSO_4$. The filtrate was evaporated and the residue purified by flash column chromatography, using a short bed (3 in) of silica gel, eluting with 9:1 EtOAc-hexanes to afford compound **4** as a colorless syrup (314 mg, 92%). R_f 0.40 (9:1 EtOAc-hexanes). $[\alpha]_D^{25}$ = - 33.6 (*c* 1.0, $CHCl_3$). Lit.[21] $[\alpha]_D^{22}$ = +32 (*c* 1.4, $CHCl_3$)*. ^1H NMR (400 MHz, $CDCl_3$) δ 1.33 (s, 3H), 1.51 (s, 3H), 1.82 (ddd, *J* = 13.5, 11.1, 4.6 Hz, 1H), 2.33 (dd, *J* = 13.5, 5.2 Hz, 1H), 4.56 (ddd, *J* = 7.1, 5.2, 1.8 Hz, 1H), 4.78 (t, *J* = 4.1 Hz, 1H), 5.94 (d, *J* = 3.5 Hz, 1H), 9.68 (d, *J* = 1.9 Hz, 1H); ^{13}C NMR (100 MHz, $CDCl_3$) δ 26.2, 26.8, 34.5, 79.9, 81.5, 106.3, 112.2, 199.9; HRMS (ESI) calcd for $C_8H_{13}O_4$ $(M+H)^+$ 173.0808, obsd 173.0807.

*We note concern over the discrepancy of optical rotations. Aldehyde **4** is not stable as evidenced by the ^1H NMR spectrum below, acquired immediately after preparation and purification.

^{1}H and ^{13}C NMR Spectra of Compounds in Scheme S1

^{1}H NMR of compound **3a**

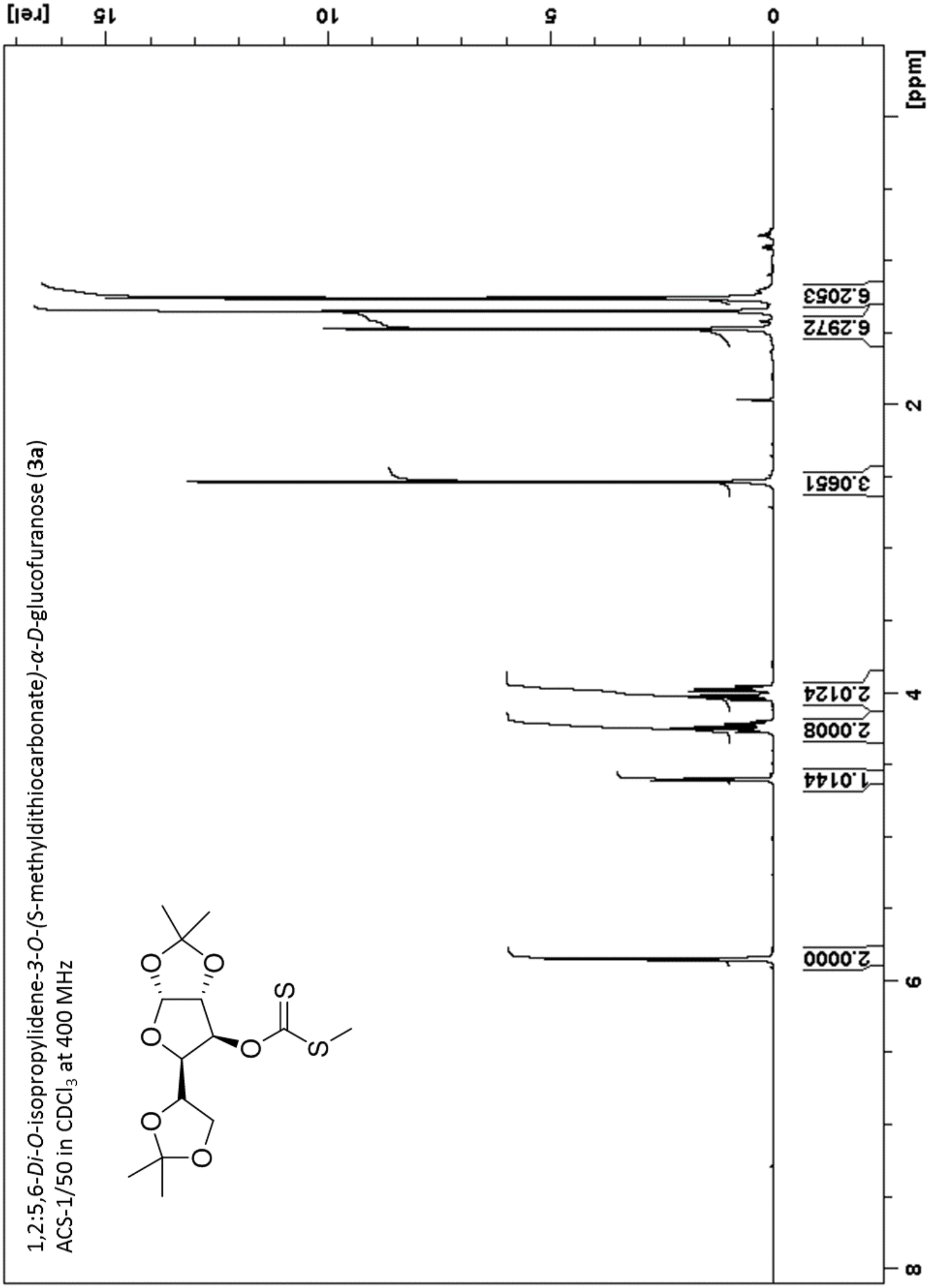

^{13}C NMR of compound **3a**

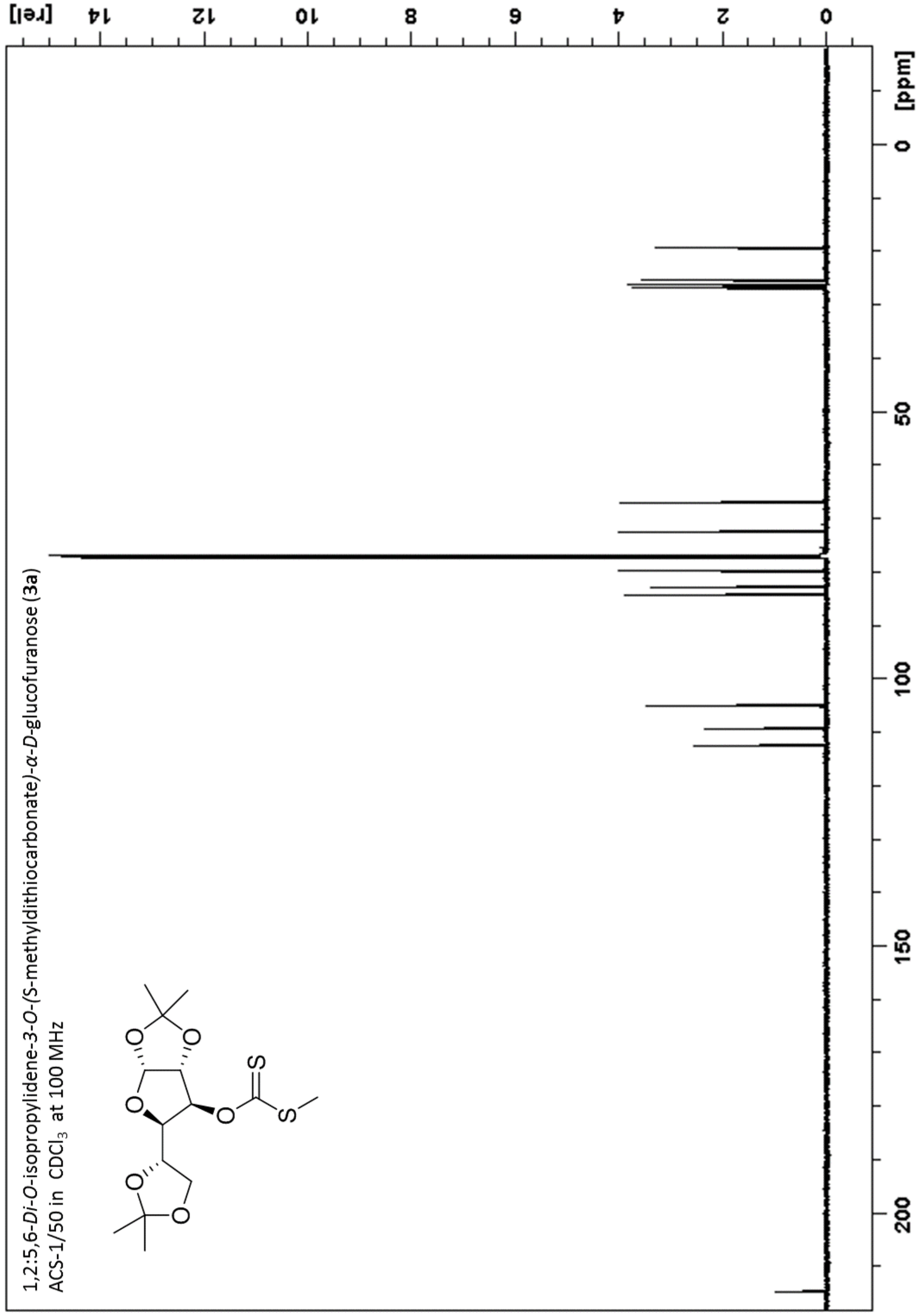

^{1}H NMR of compound **3b**

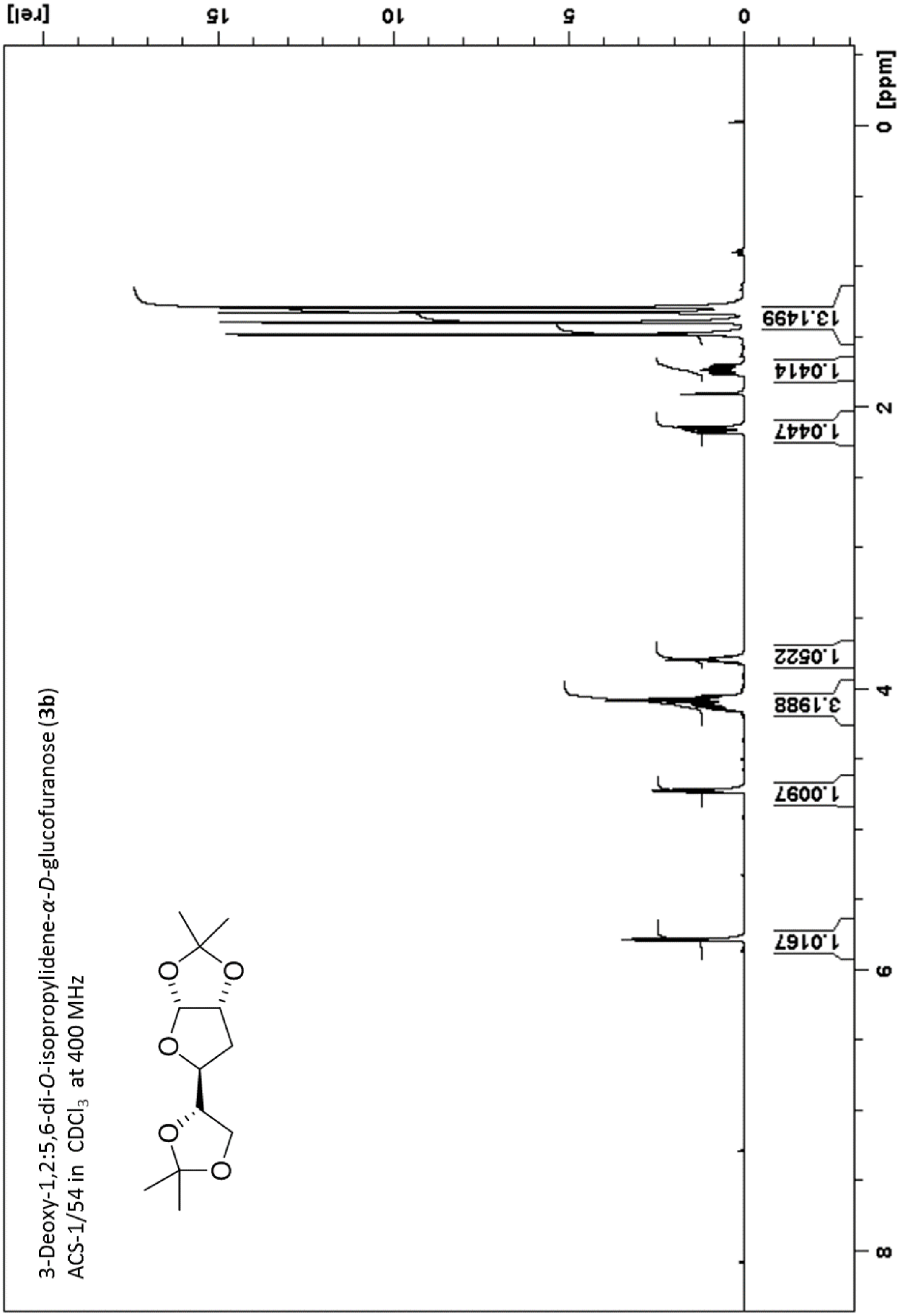

^{13}C NMR of compound **3b**

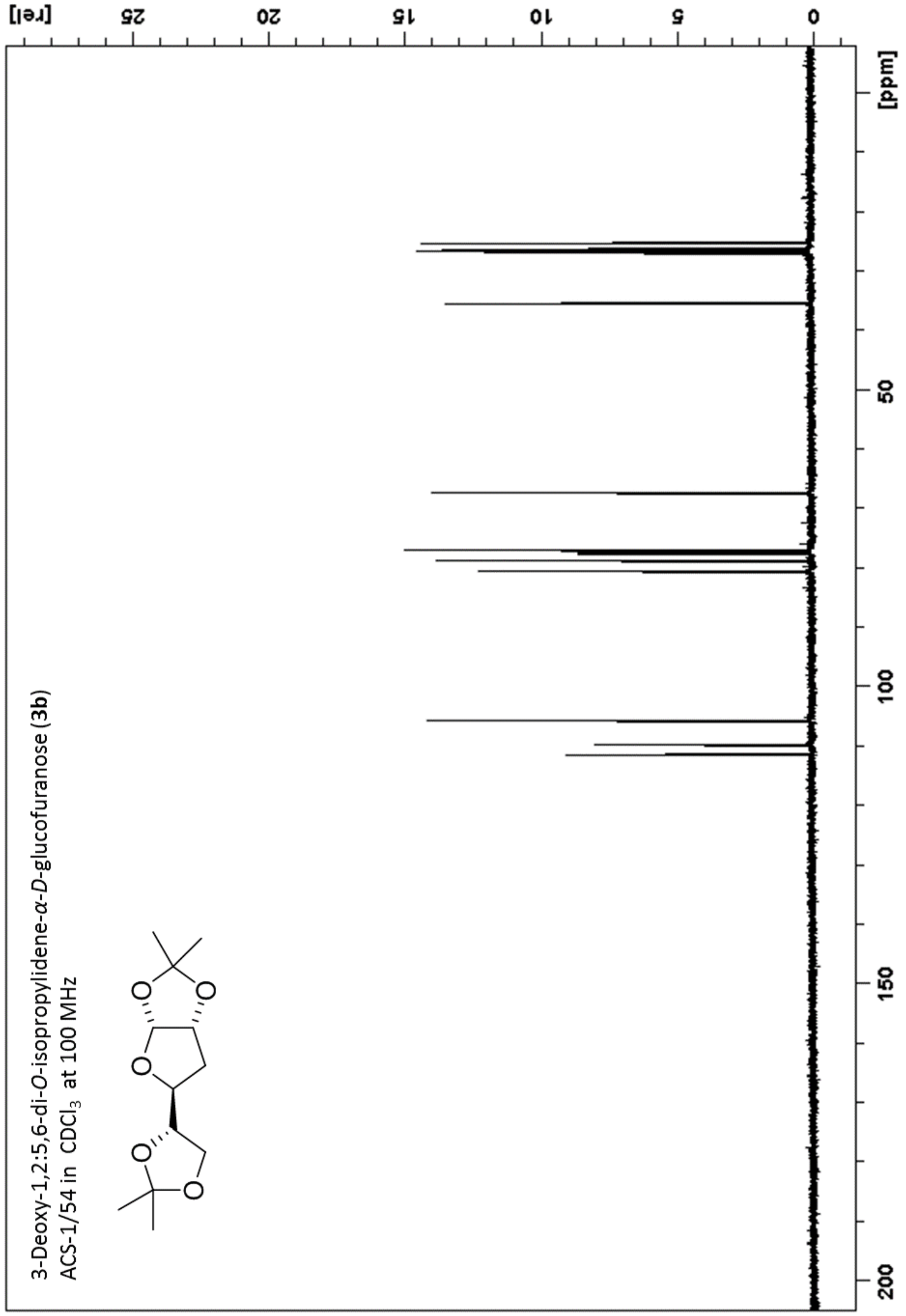

^{1}H NMR of compound **3c**

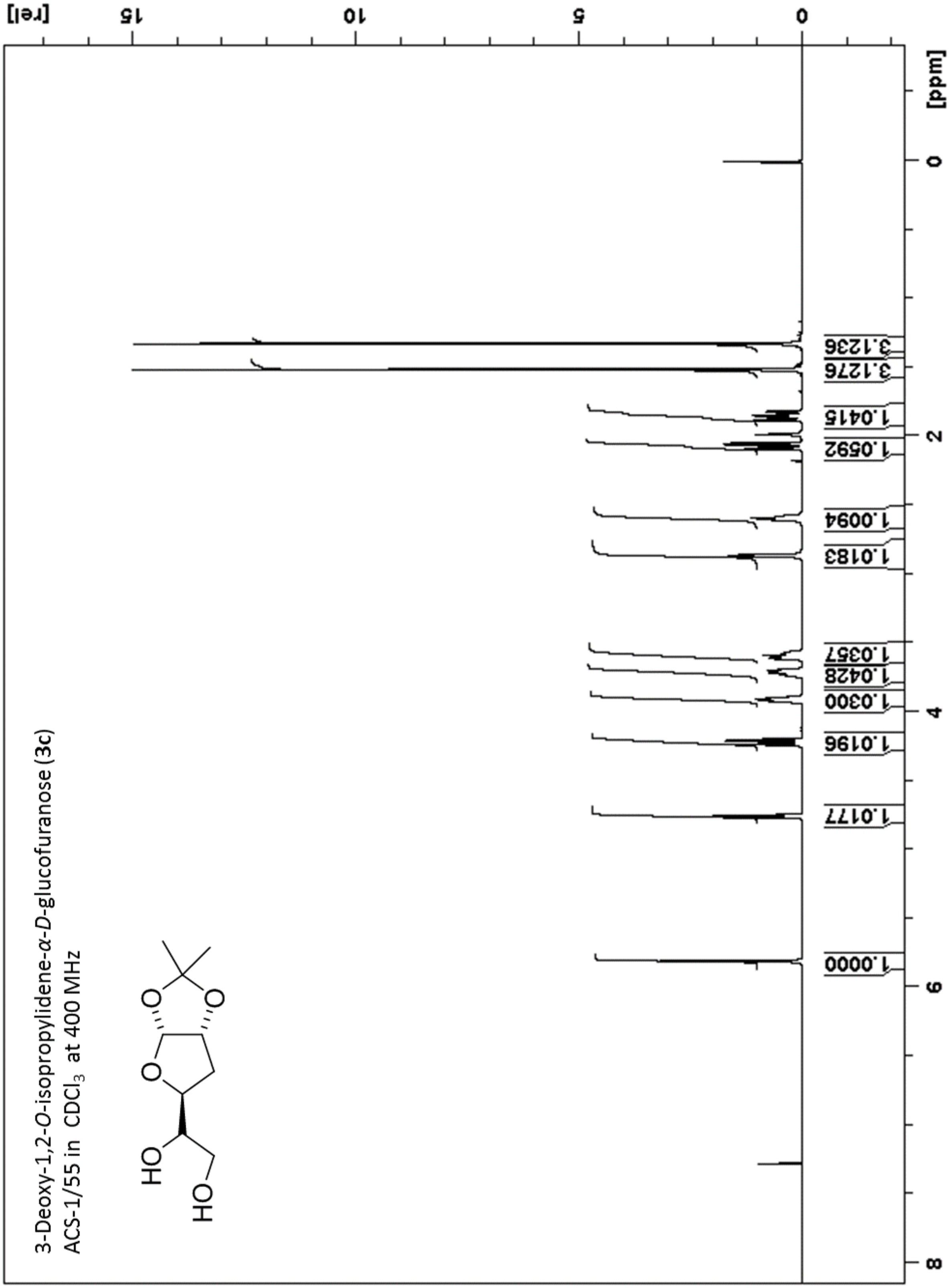

^{13}C NMR of compound **3c**

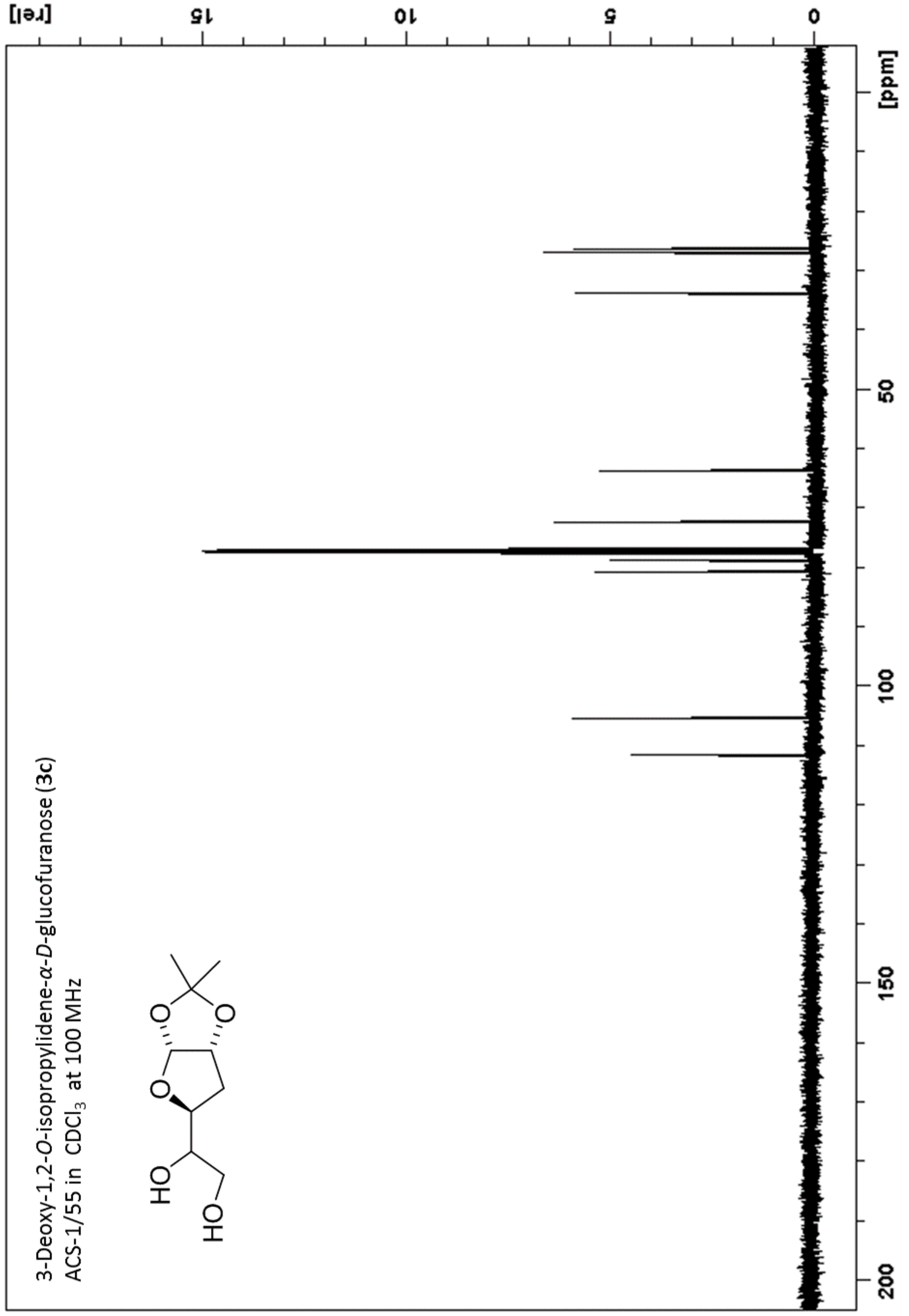

^{1}H NMR of compound **4**

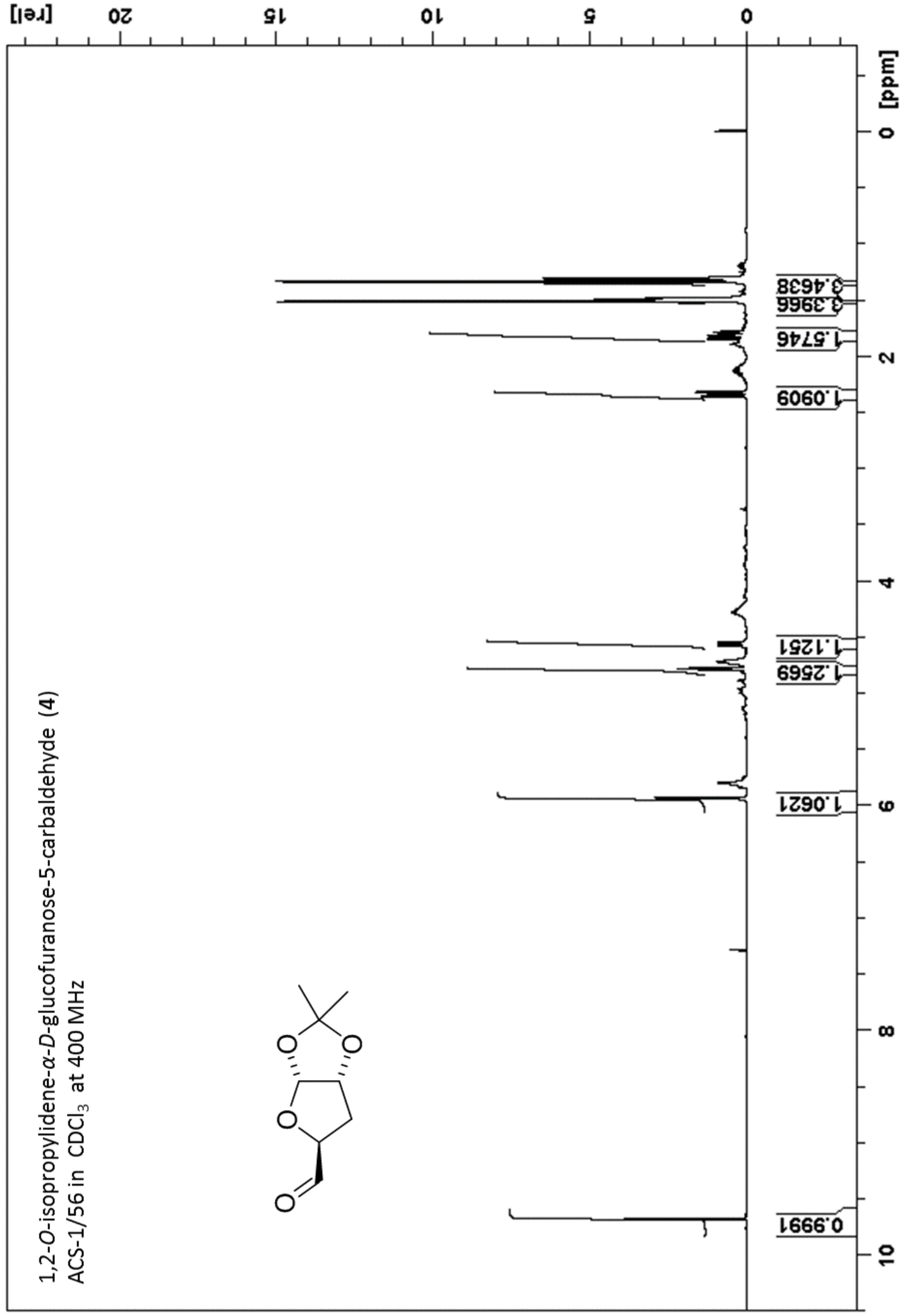

^{13}C NMR of compound **4**

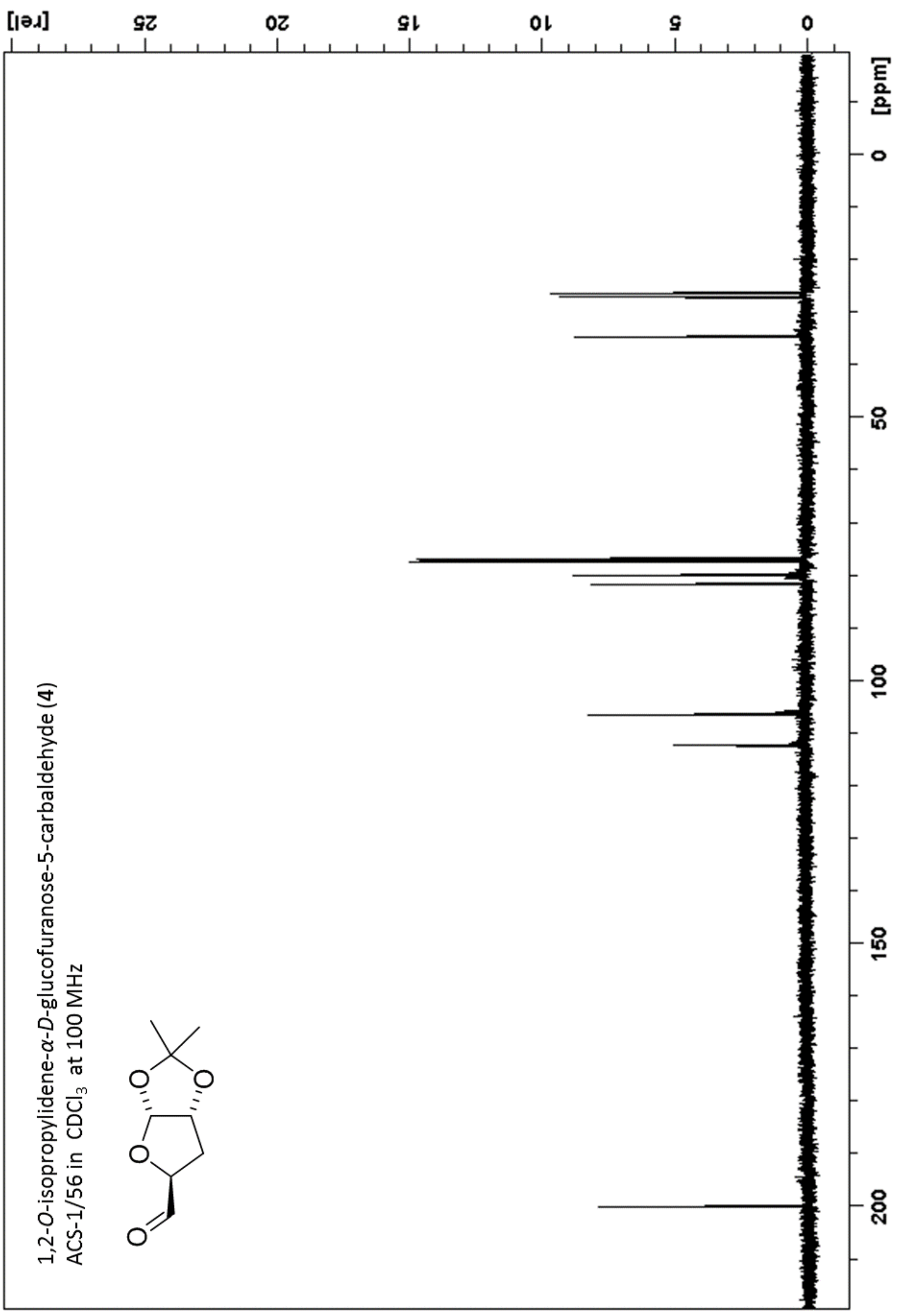

^{1}H and ^{13}C NMR Spectra of Compounds in Scheme 1

^{1}H NMR of compound **6**

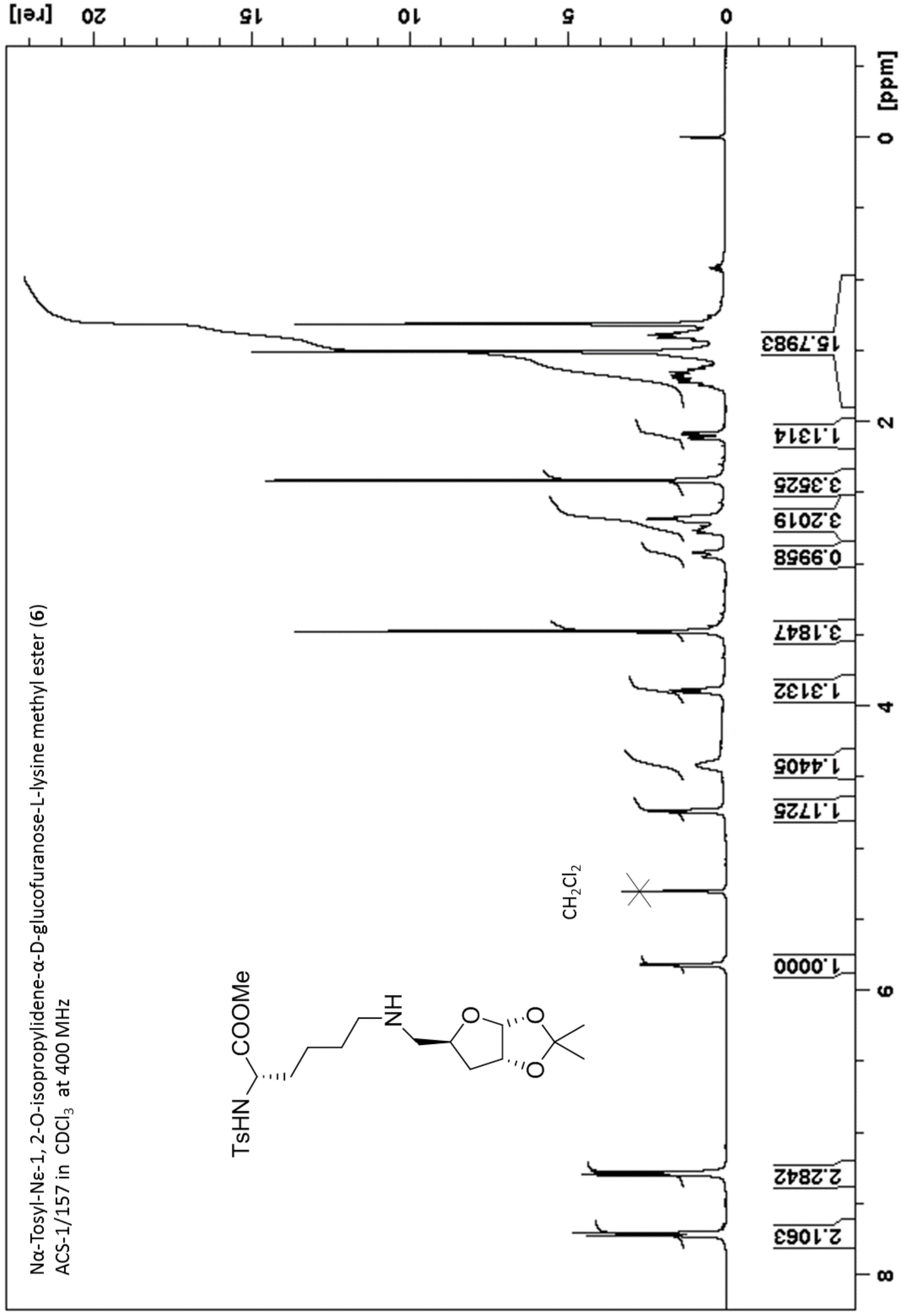

^{13}C NMR of compound **6**

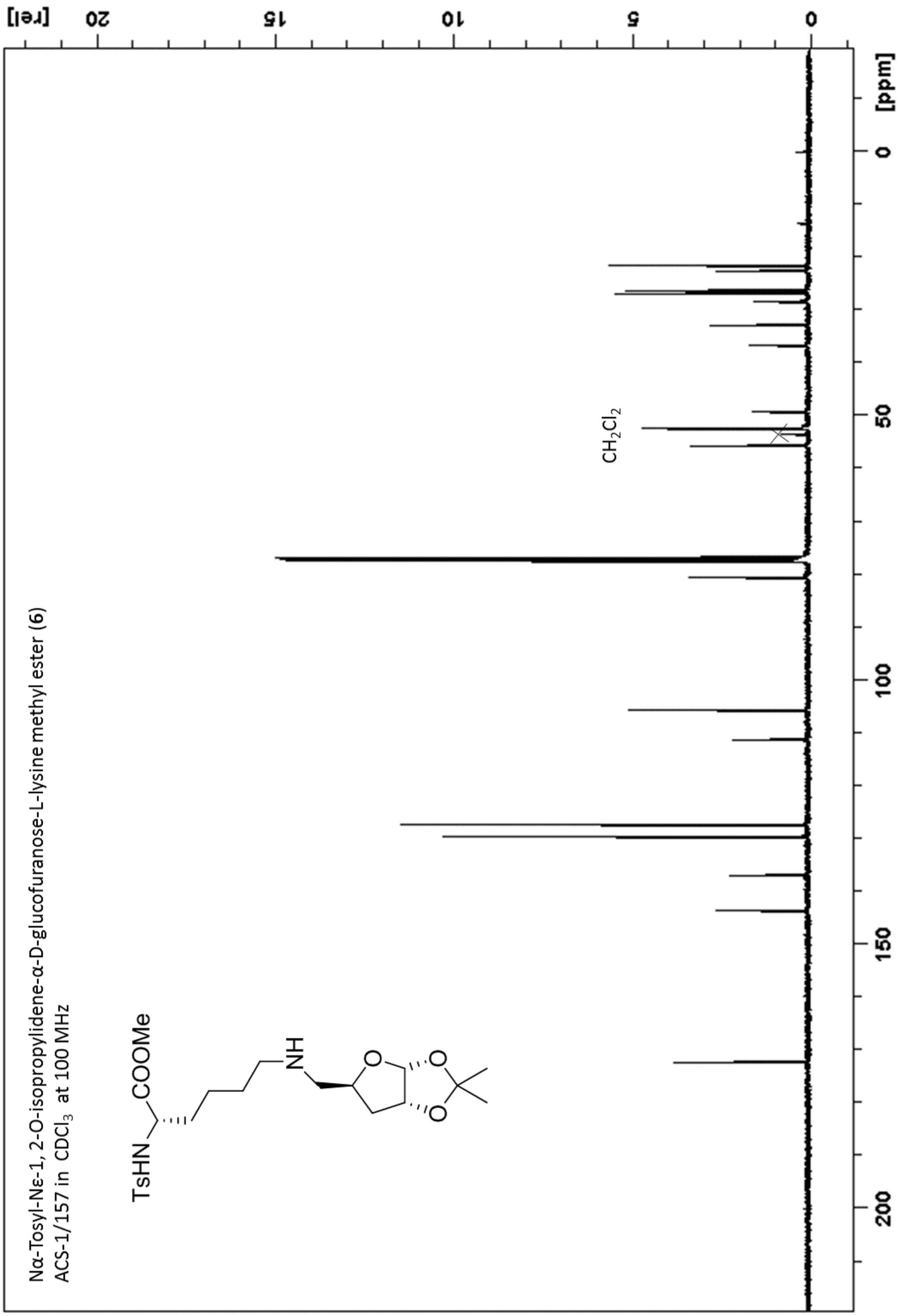

^{1}H NMR of compound **2**

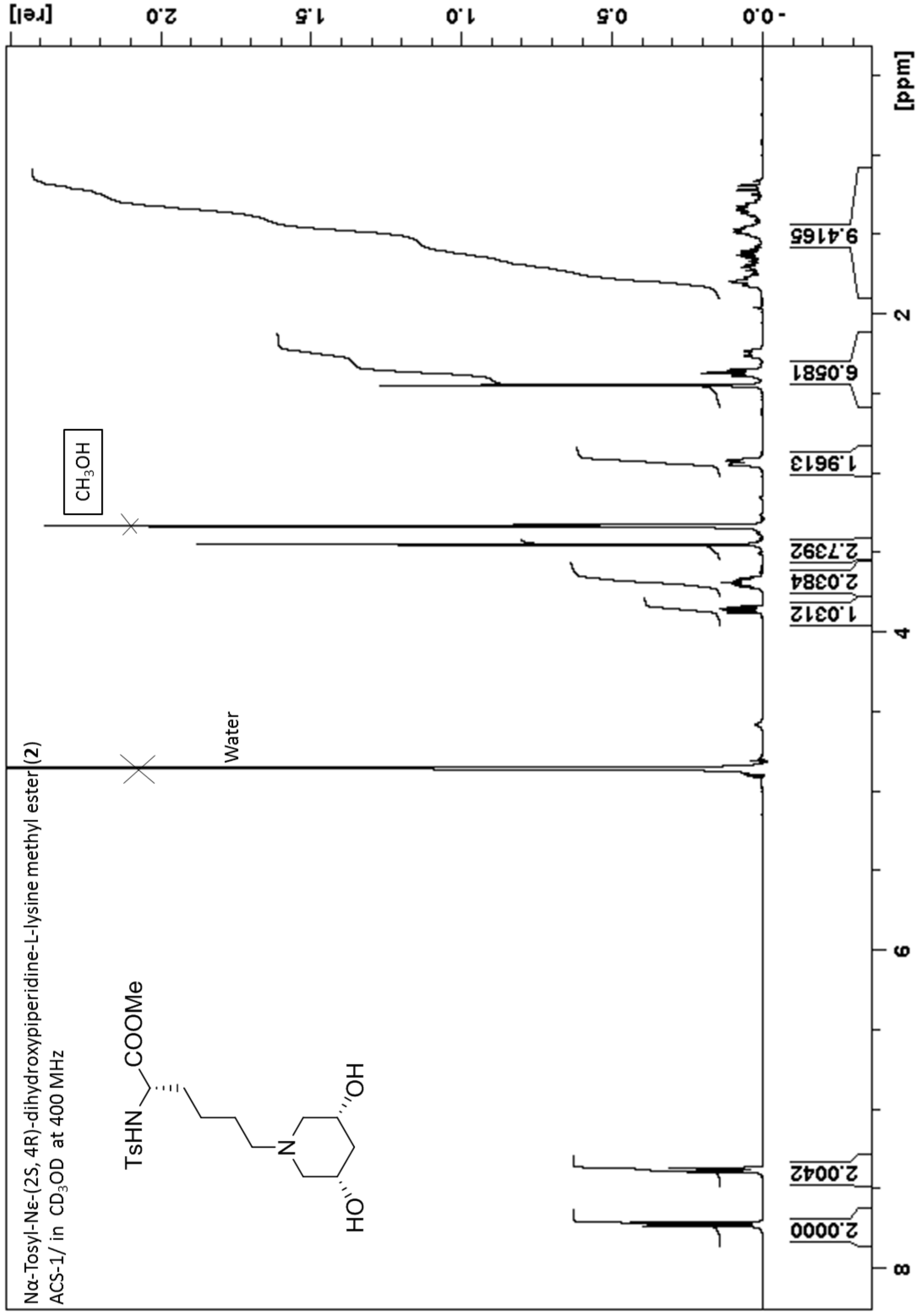

^{13}C NMR of compound **2**

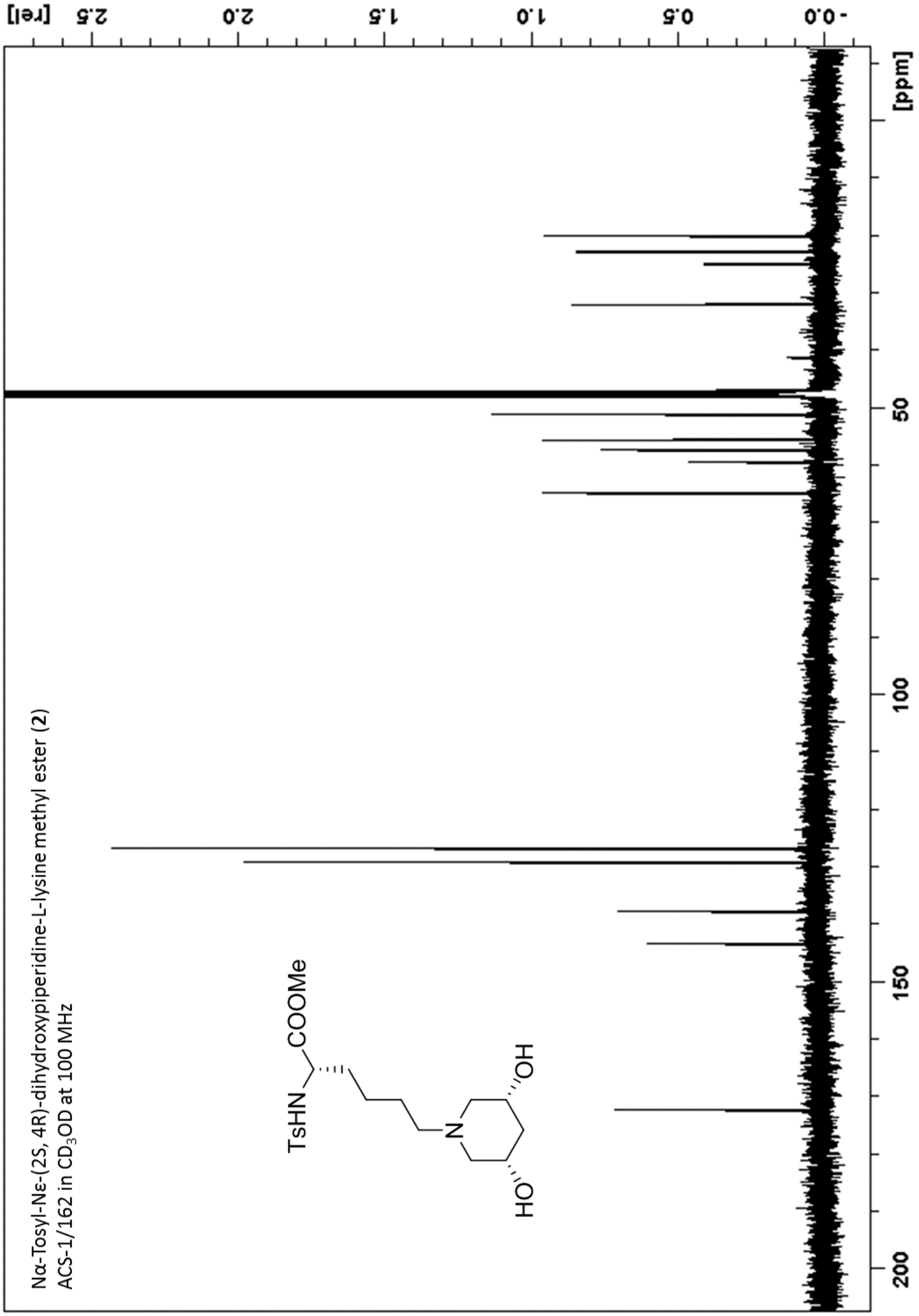

^{1}H and ^{13}C NMR Spectra of Compounds in Scheme 2

^{1}H NMR of compound **10a**

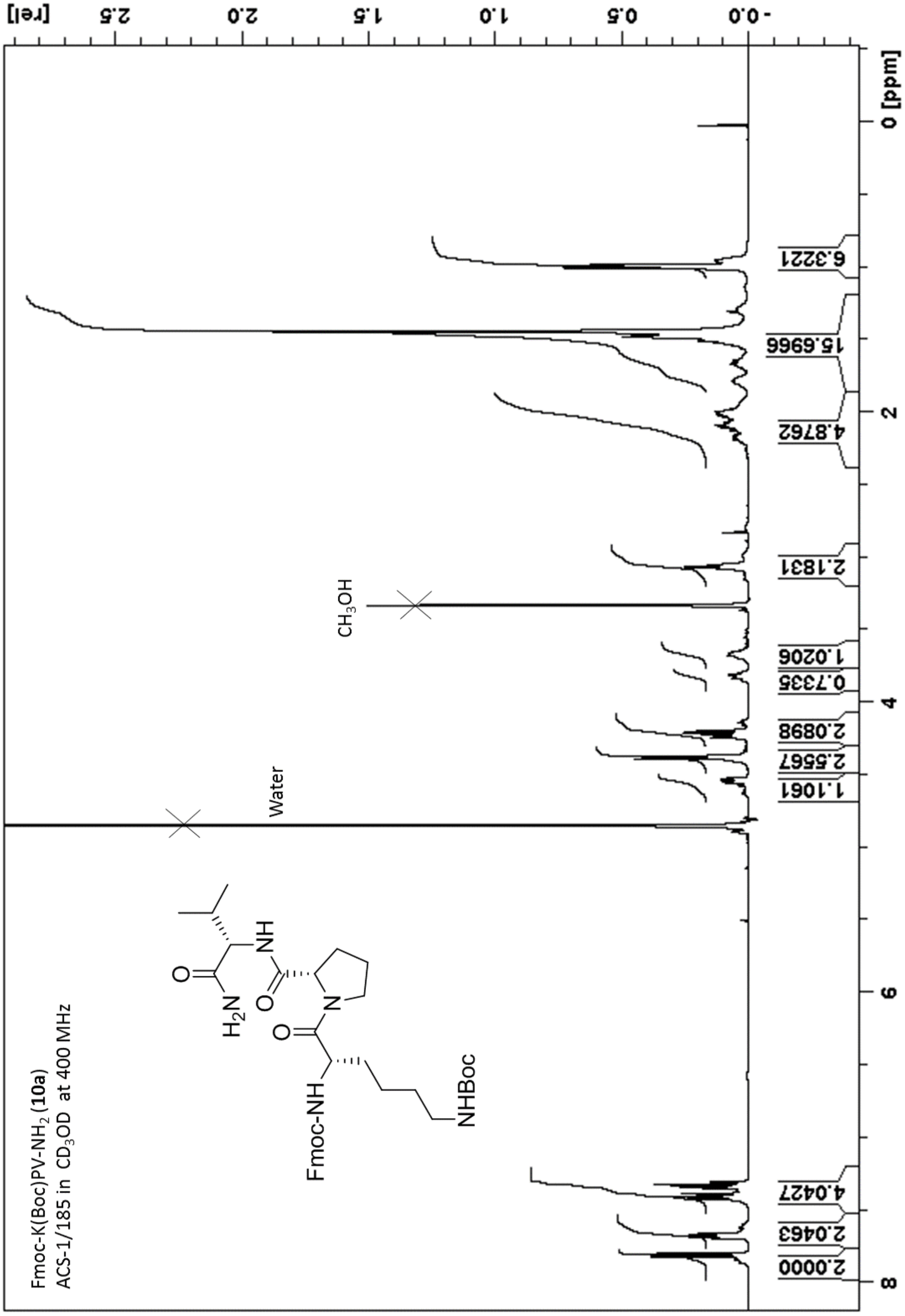

^{13}C NMR of compound **10a**

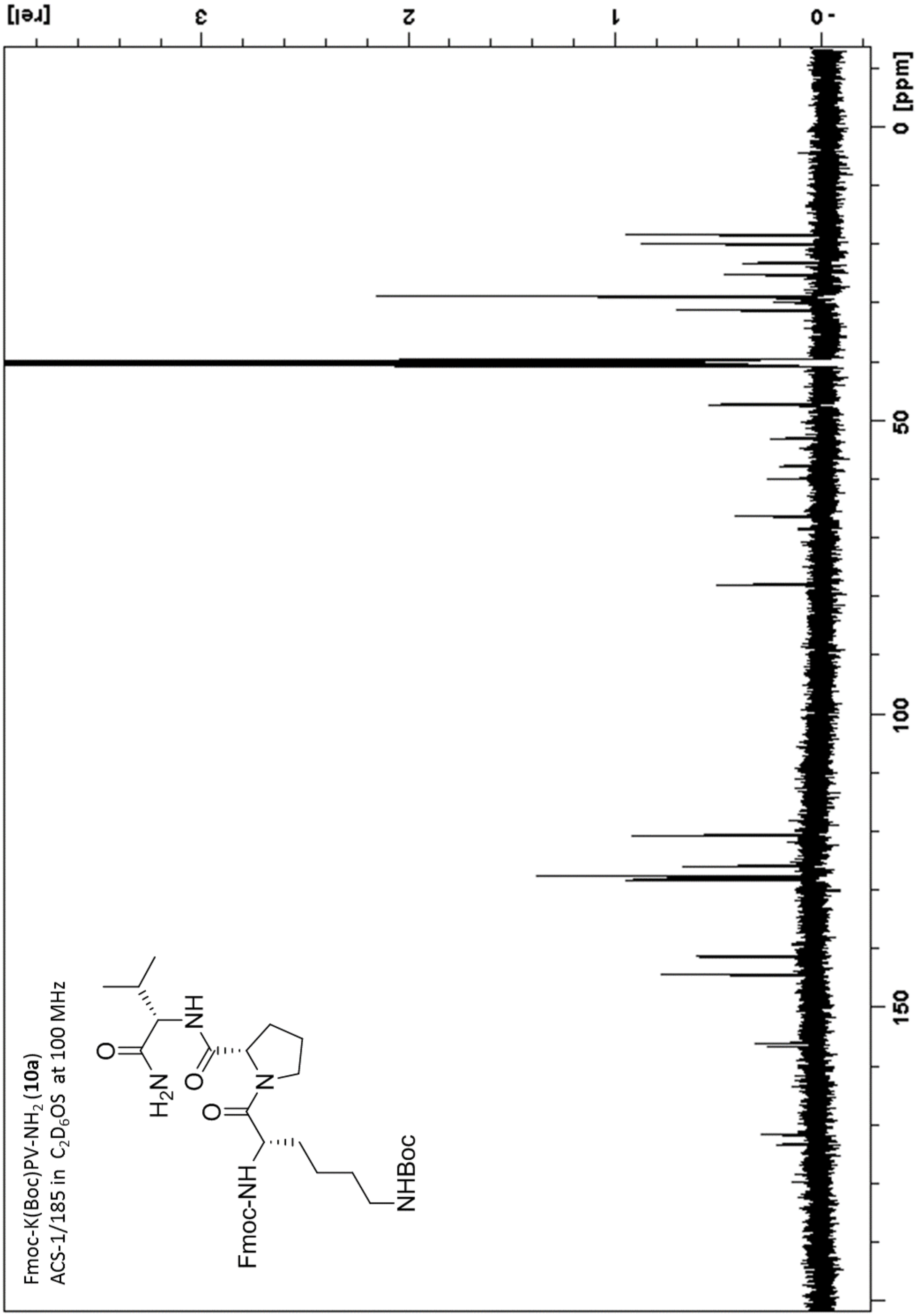

^{1}H NMR of compound **10b**

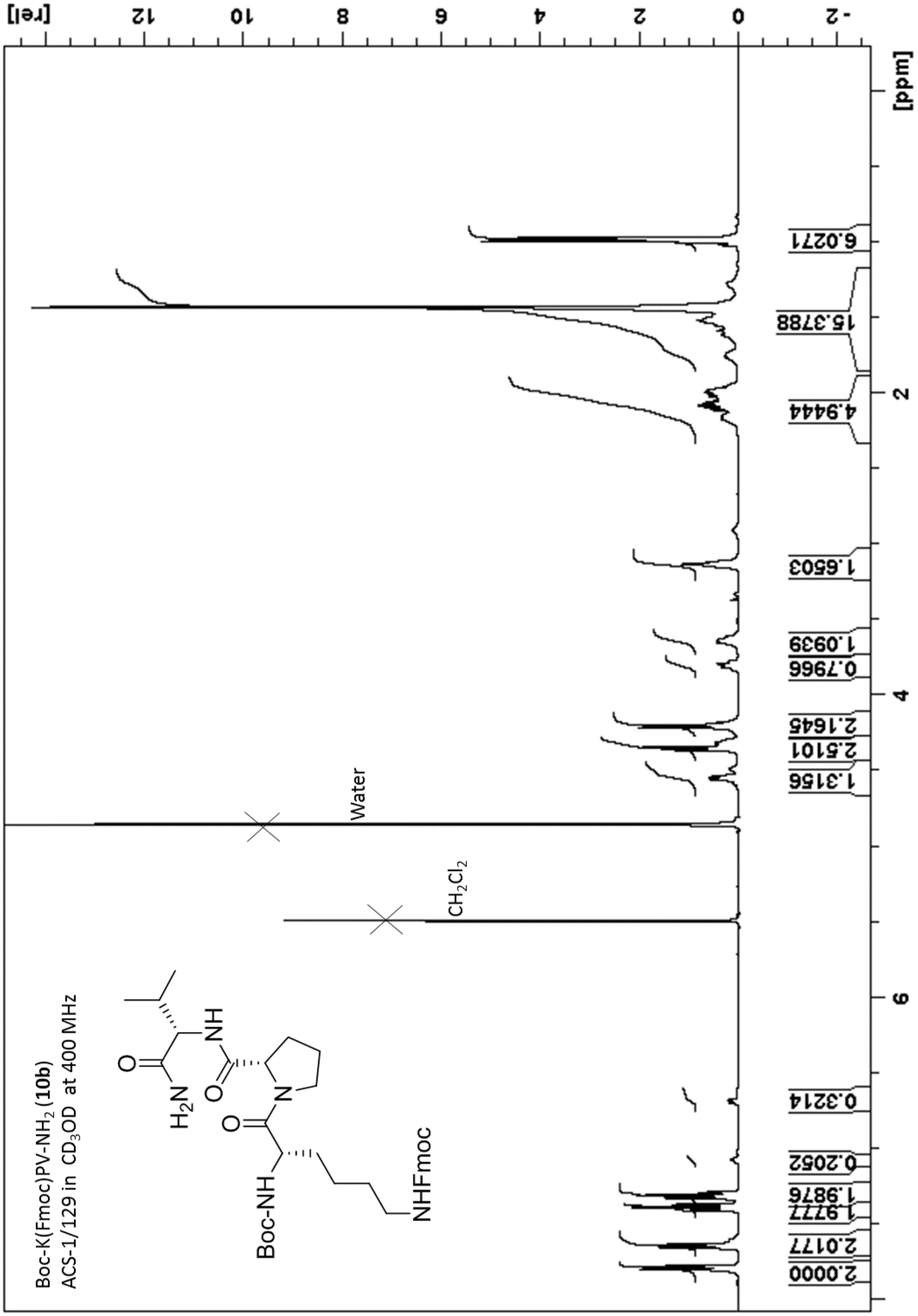

^{13}C NMR of compound **10b**

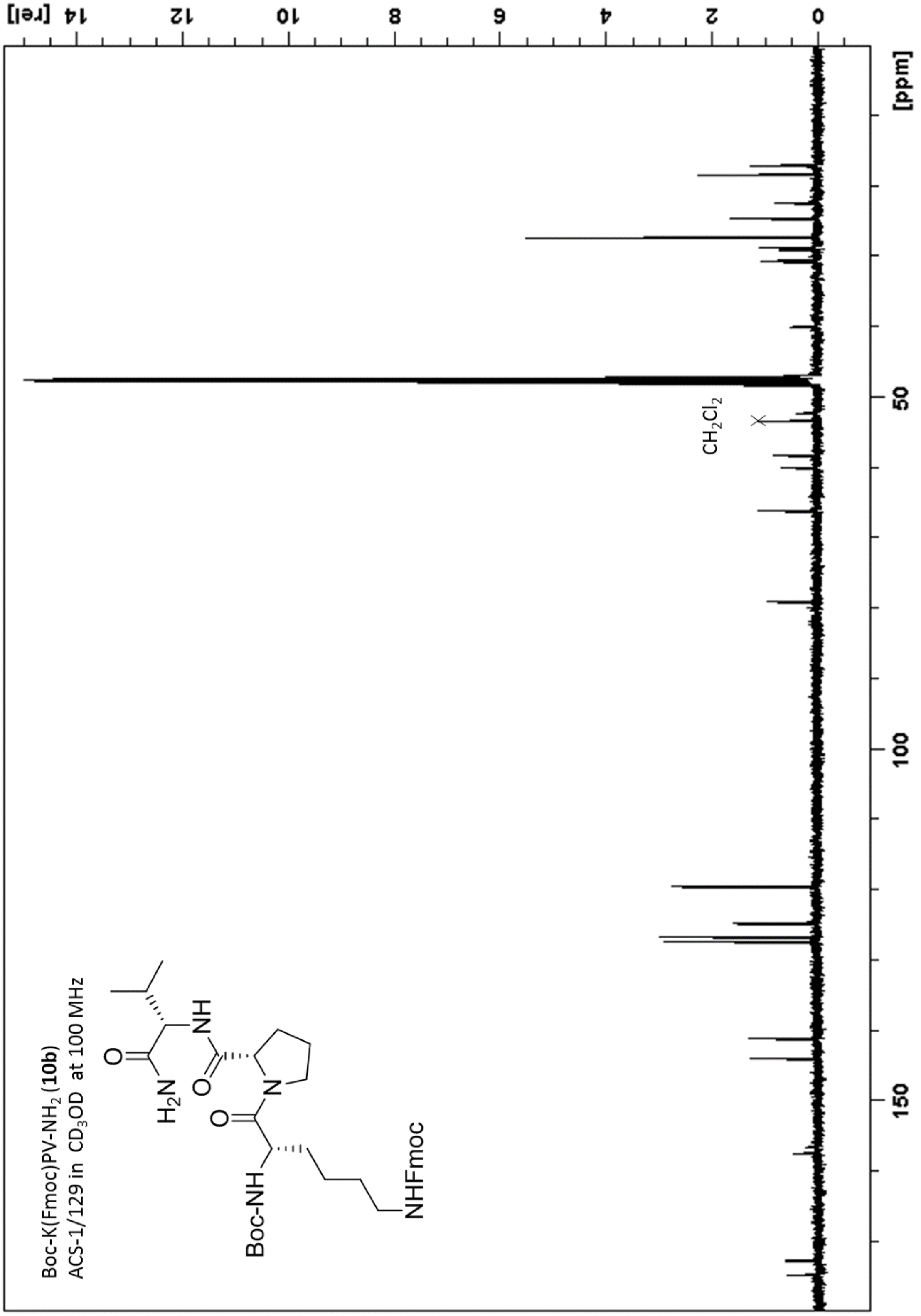

^{1}H and ^{13}C NMR Spectra of Compounds in Scheme 3

^{1}H NMR of compound **11a**

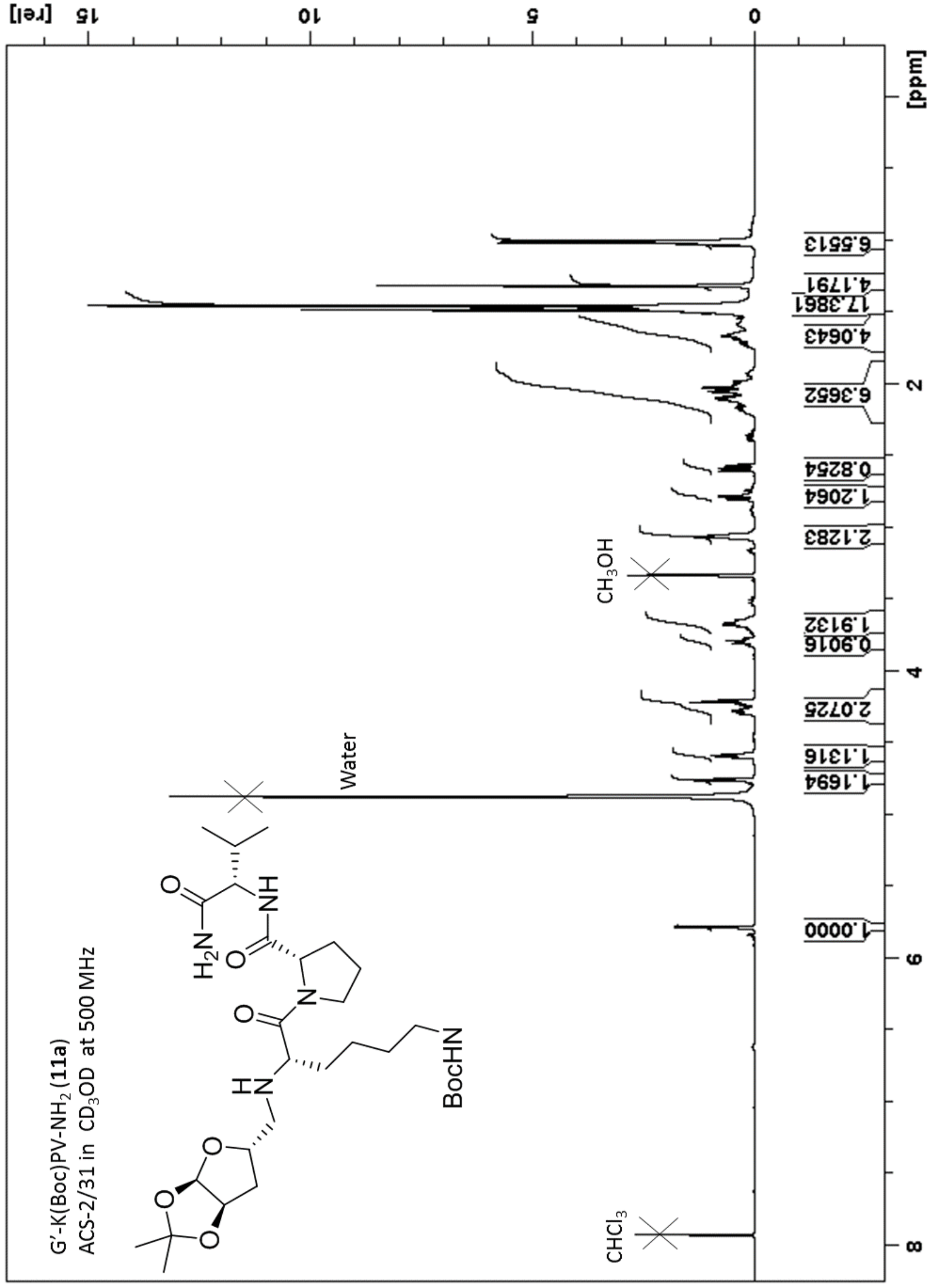

^{13}C NMR of compound **11a**

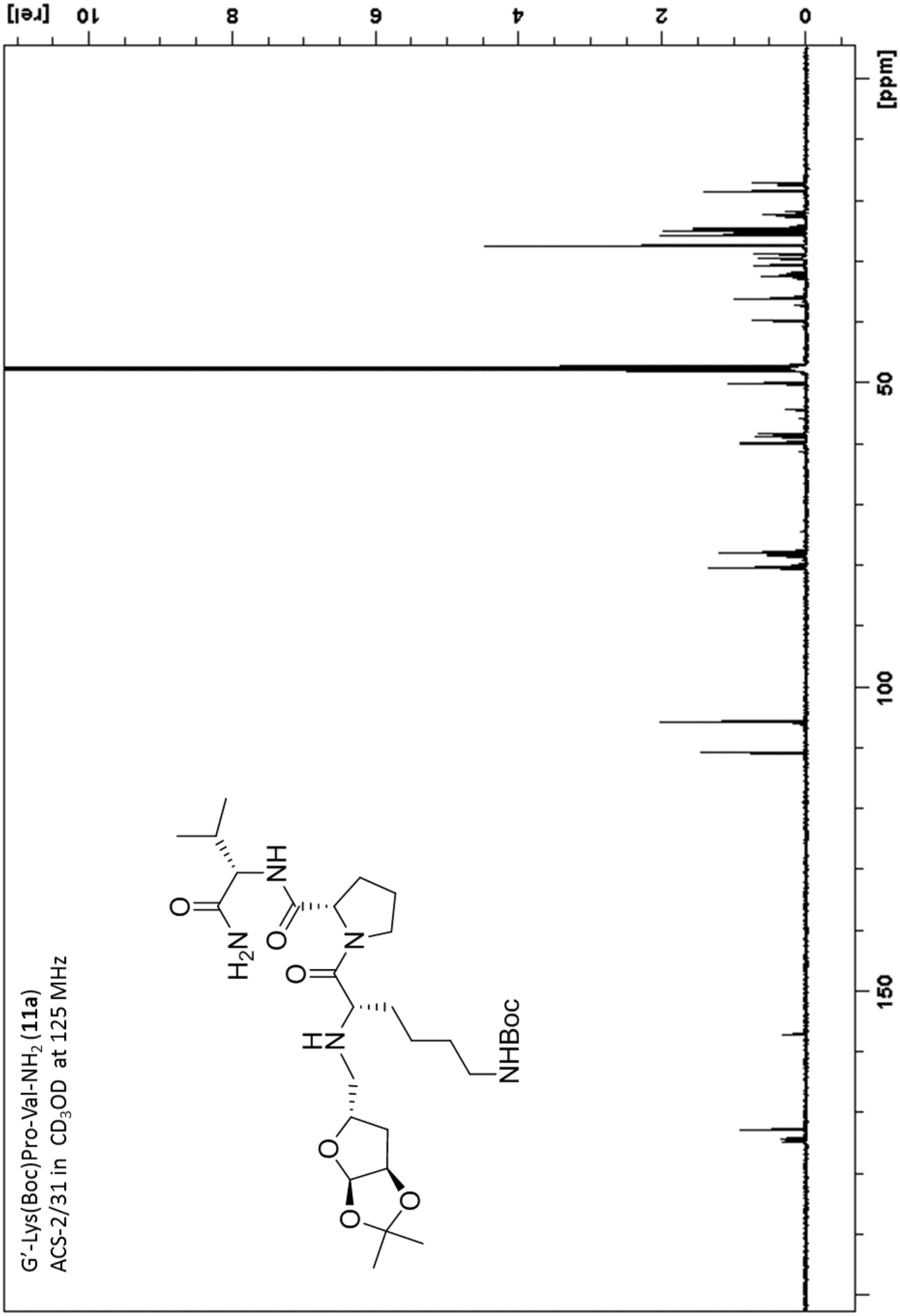

^{1}H NMR of compound **11b**

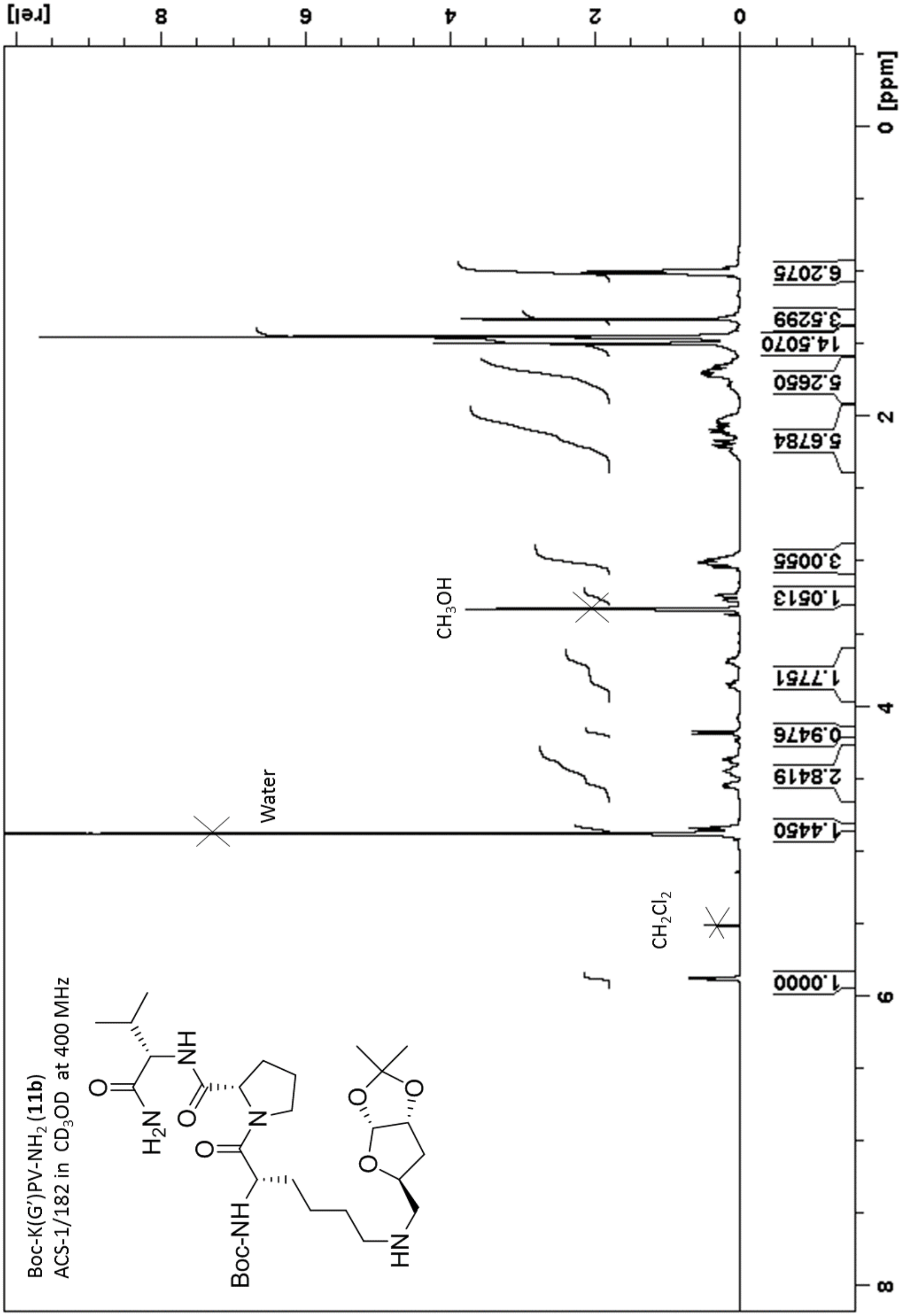

^{13}C NMR of compound **11b**

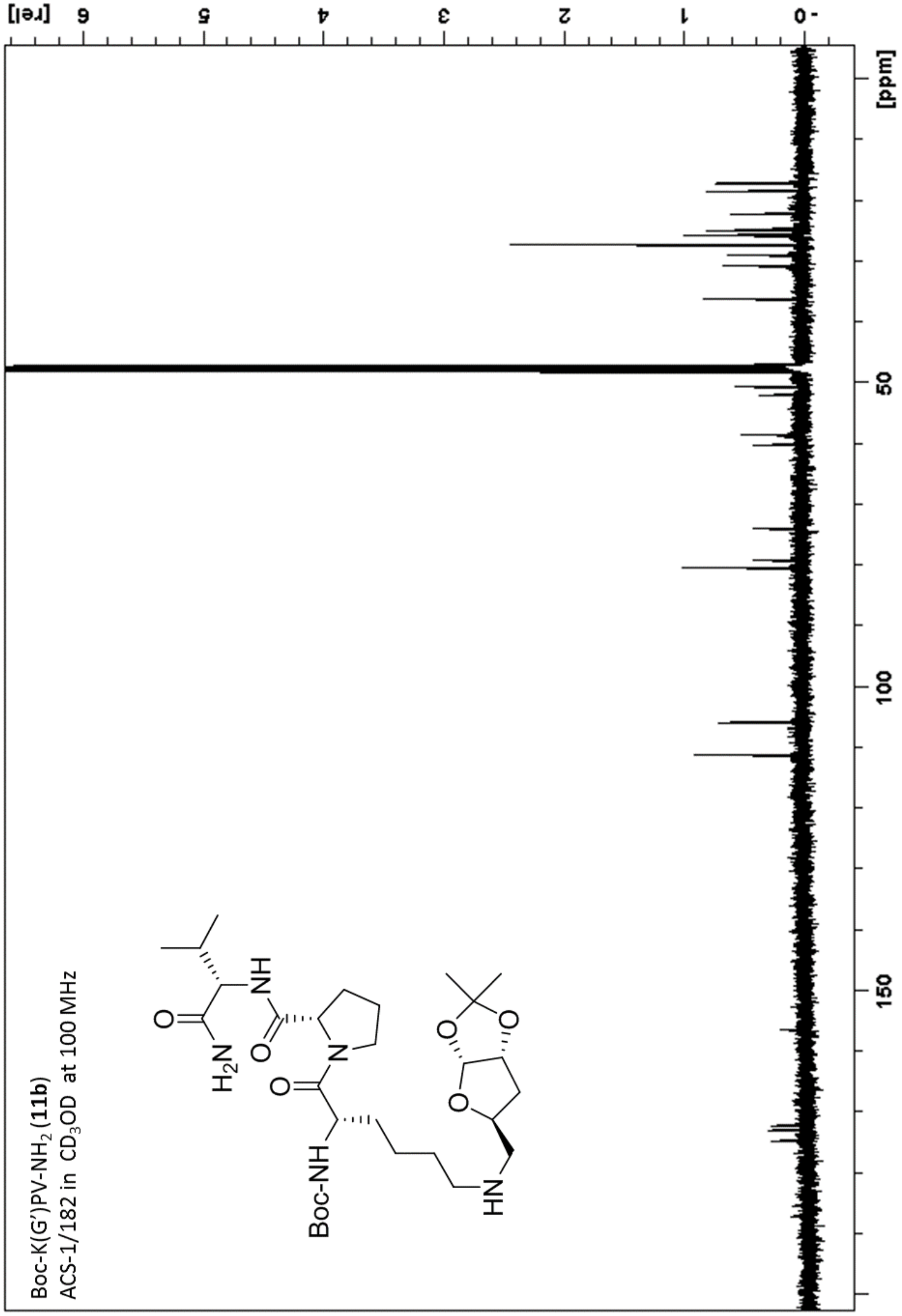

^{1}H NMR of compound **12a**

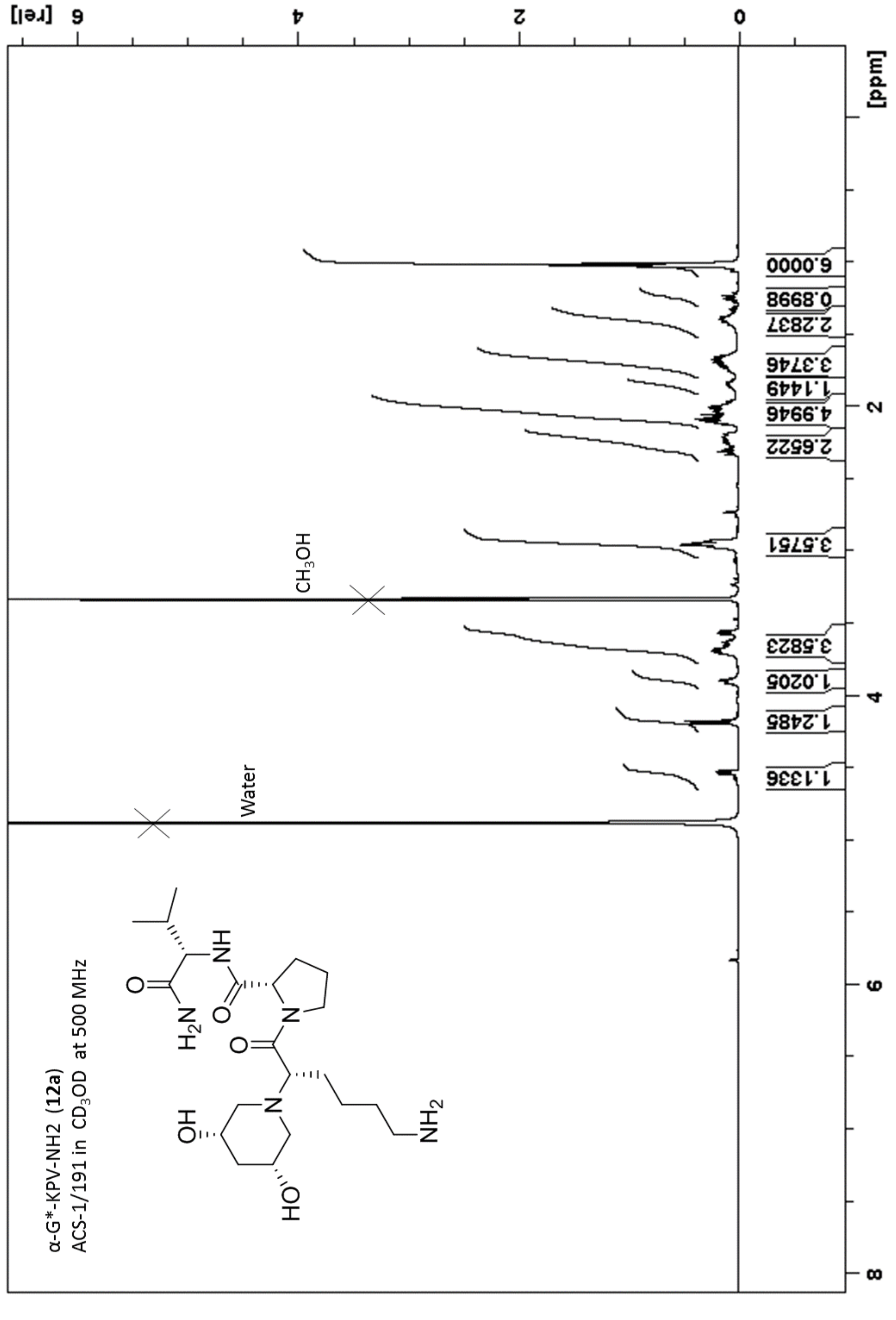

^{13}C NMR of compound **12a**

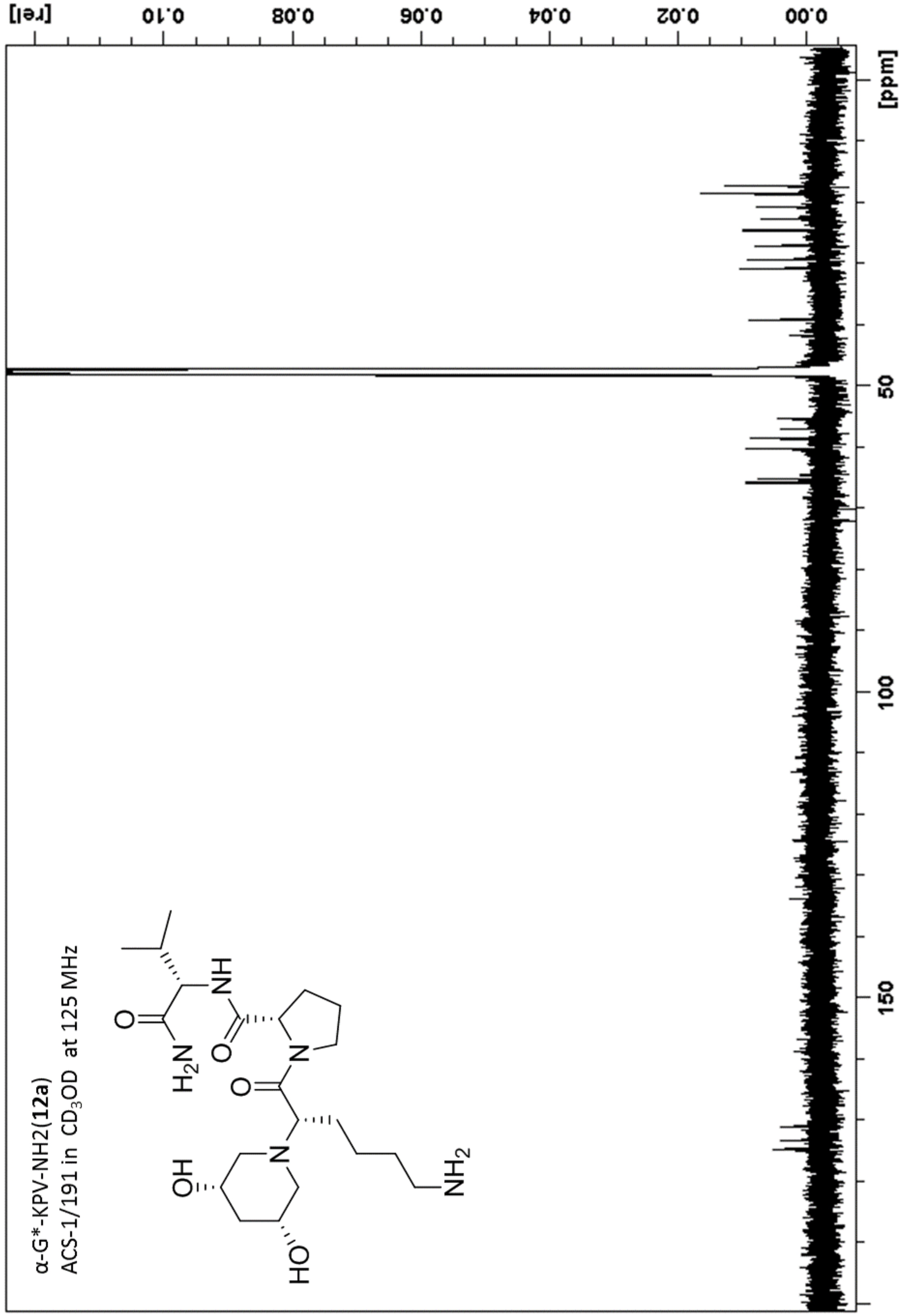

^{1}H NMR of compound **12b**

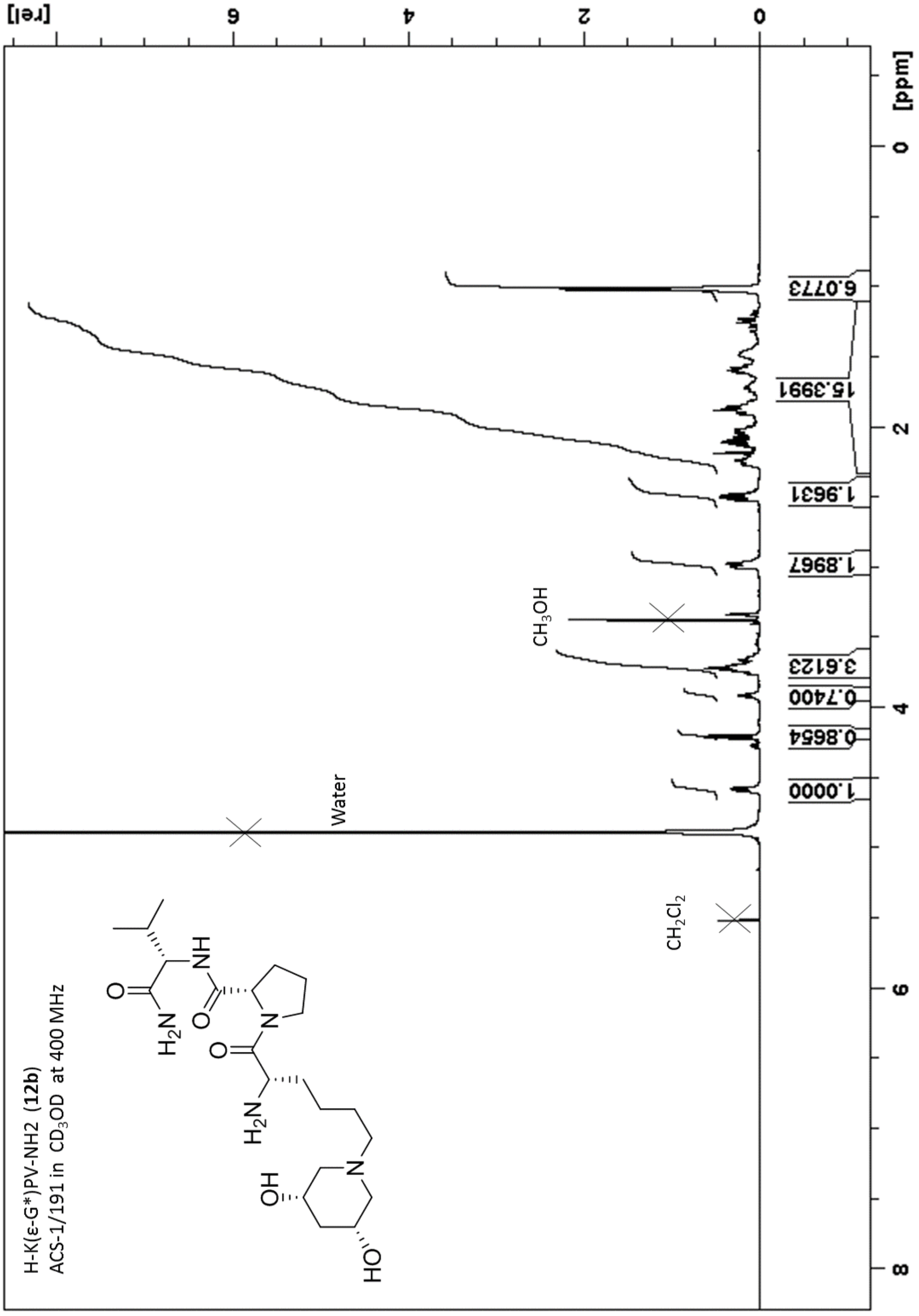

^{13}C NMR of compound **12b**

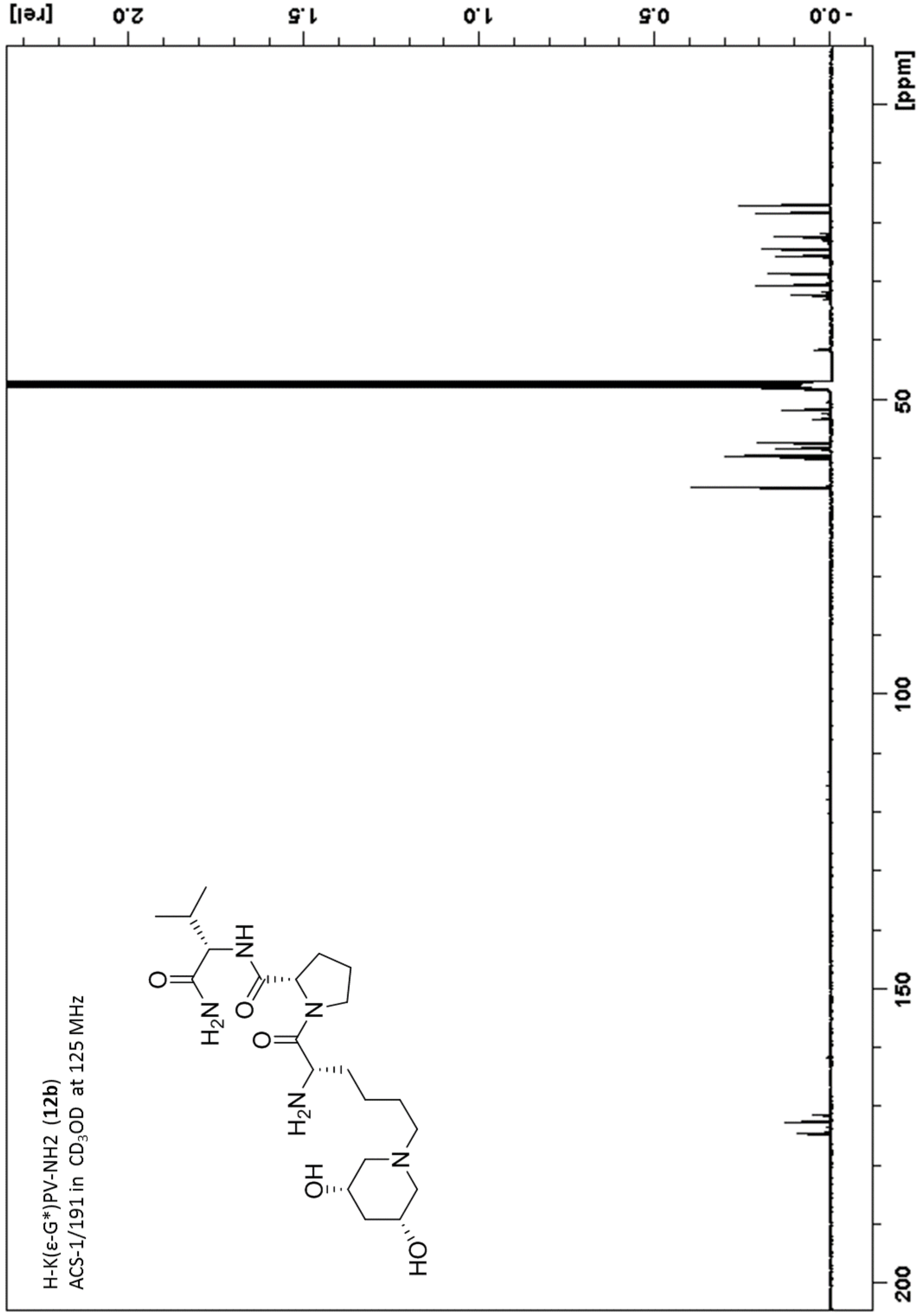

^{1}H NMR of compound **11c**

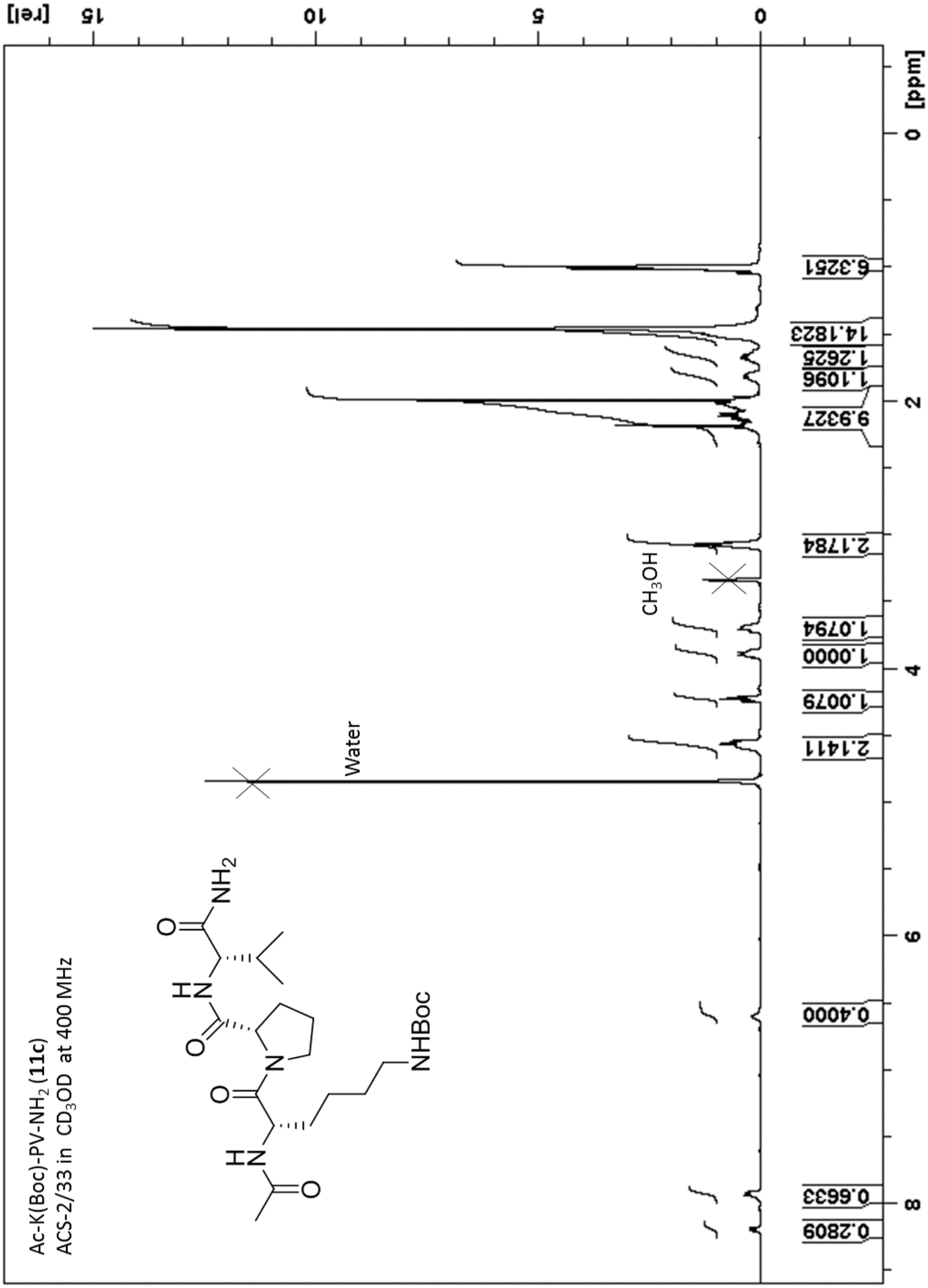

^{13}C NMR of compound **11c**

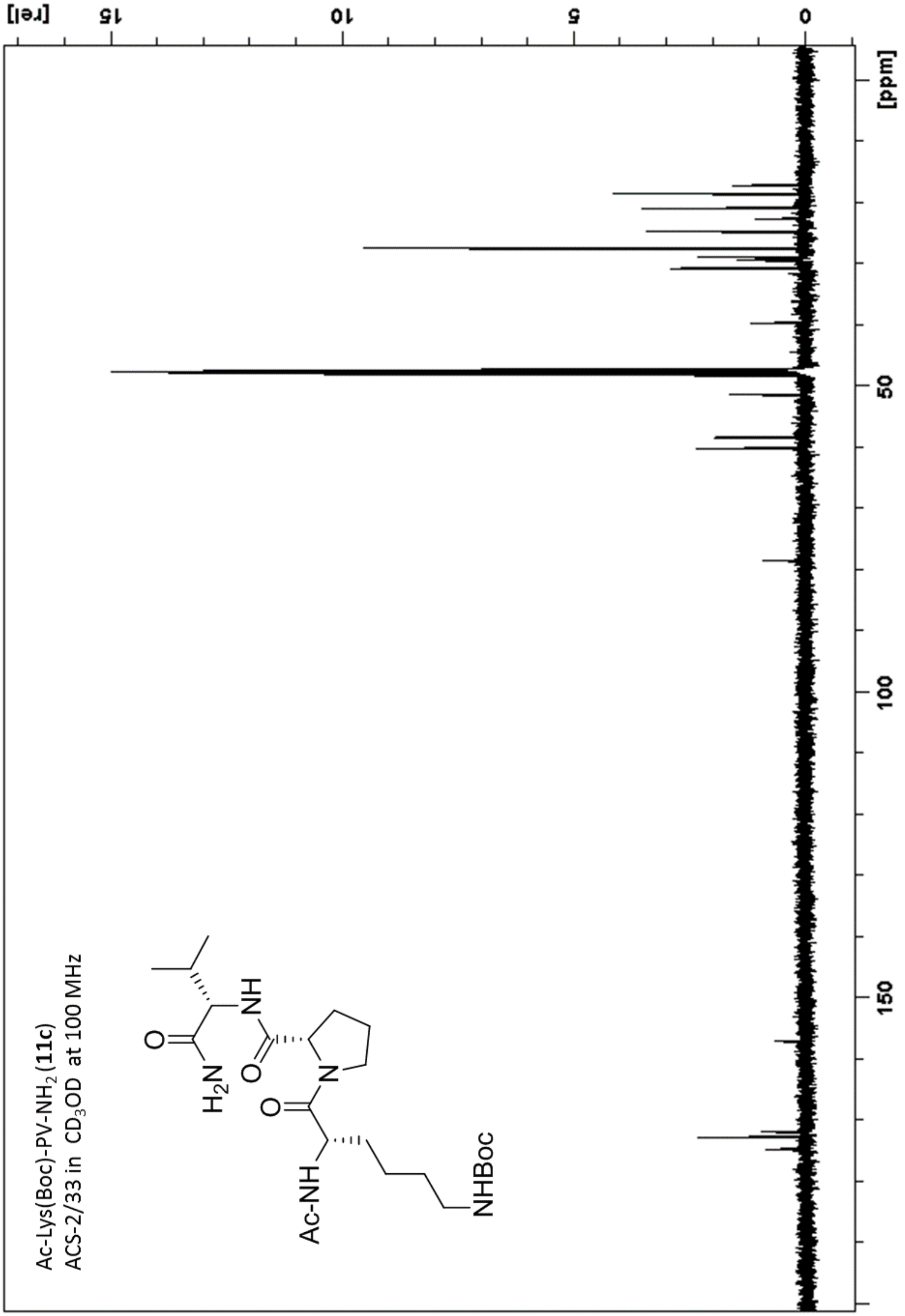

^{1}H NMR of compound **12c**

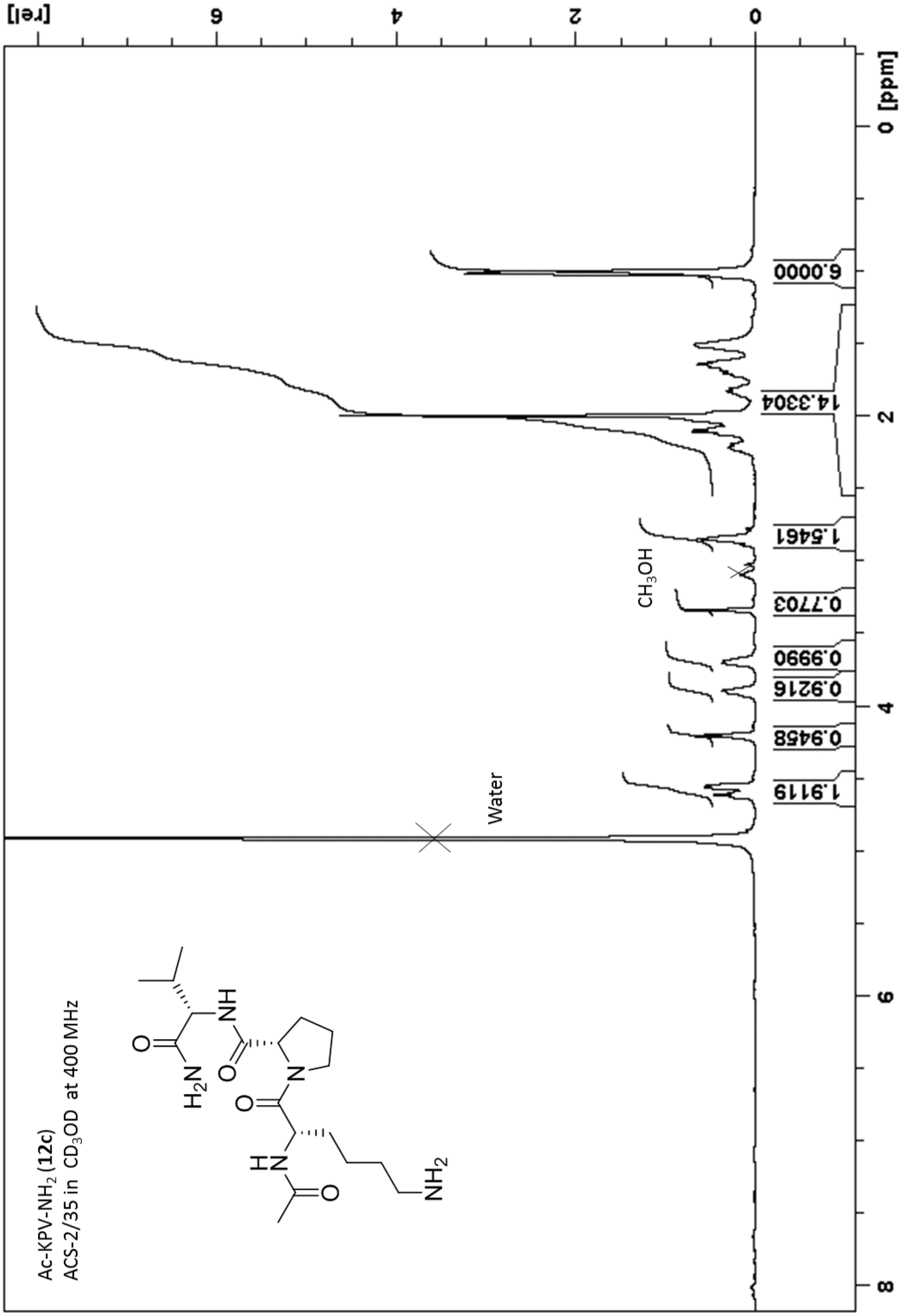

^{13}C NMR of compound **12c**

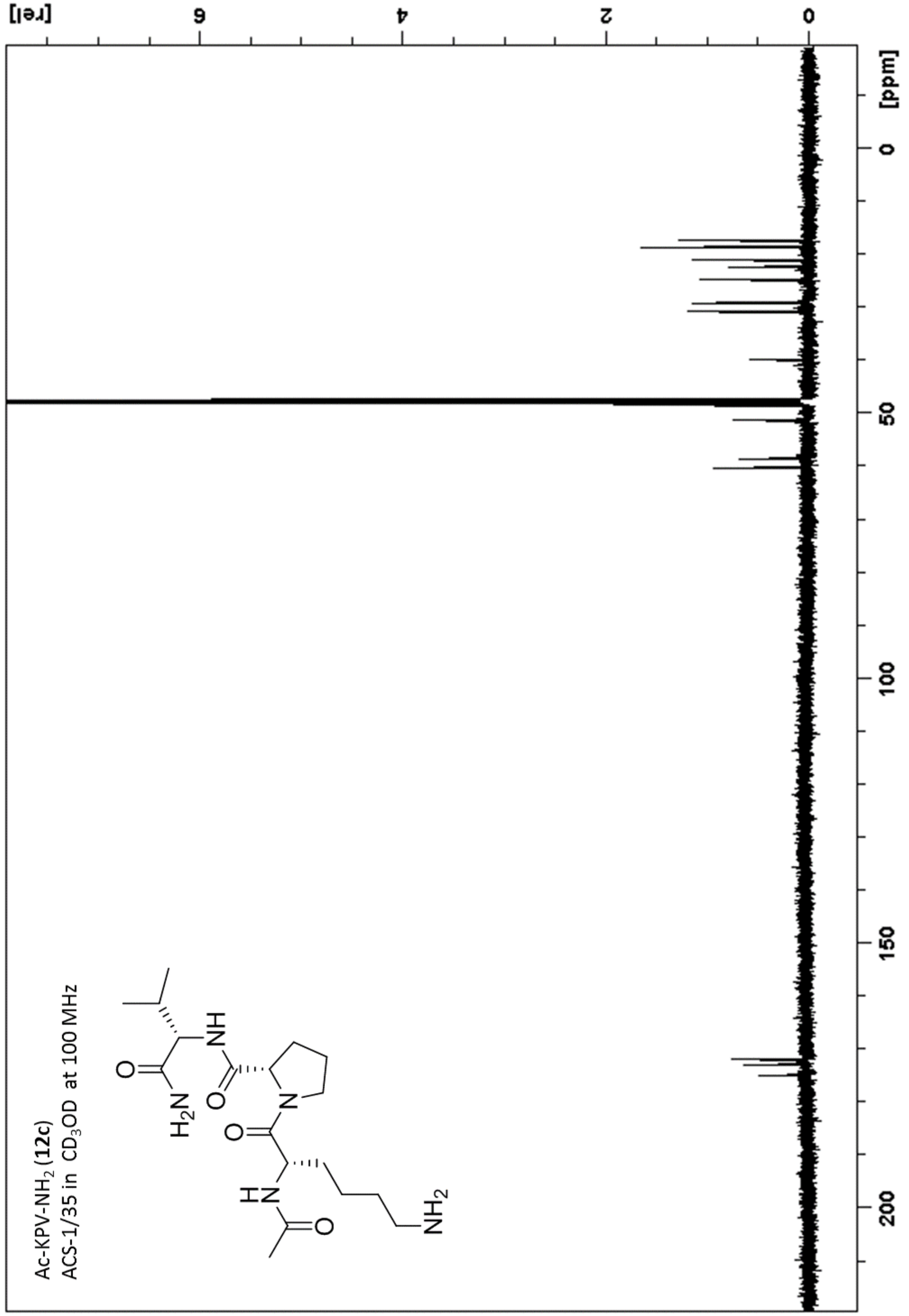

Compound 2

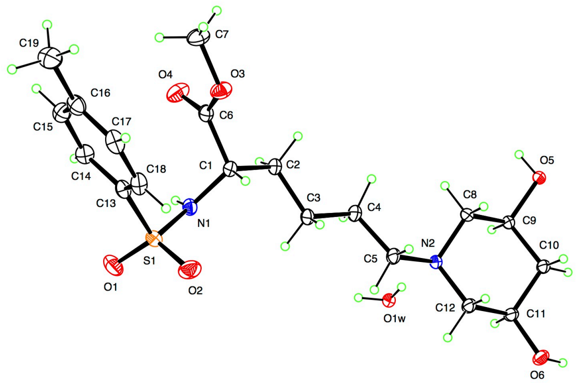

Details for X-Ray Structure Determination

Computing details

Data collection: Bruker *APEX2*; cell refinement: Bruker *SAINT*; data reduction: Bruker *SAINT*; program(s) used to solve structure: *SHELXS97* (Sheldrick, 2008); program(s) used to refine structure: *SHELXL97* (Sheldrick, 2008); molecular graphics: *ORTEP-3 for Windows* (Farrugia, 2012); software used to prepare material for publication: *SHELXL97* (Sheldrick, 2008).

(Songok1)

Crystal data

$C_{19}H_{30}N_2O_6S{\cdot}H_2O$
M_r = 432.53
Orthorhombic, $P2_12_12_1$
Hall symbol: P 2ac 2ab
a = 7.3403 (2) Å
b = 7.9885 (2) Å
c = 36.4564 (10) Å
V = 2137.73 (10) Å^3
Z = 4
F(000) = 928
D_x = 1.344 Mg m^{-3}
Mo $K\alpha$ radiation, λ = 0.71073 Å
Cell parameters from 9374 reflections
θ = 2.6–31.1°
μ = 0.19 mm^{-1}
T = 100 K
Fragment, colourless
0.27 × 0.24 × 0.17 mm

Data collection

Bruker Kappa APEX-II DUO diffractometer
Radiation source: fine-focus sealed tube
TRIUMPH curved graphite monochromator
φ and ω scans
Absorption correction: multi-scan *SADABS* (Sheldrick, 2004)
T_{min} = 0.896, T_{max} = 0.968
43780 measured reflections
7103 independent reflections
6772 reflections with $I > 2\sigma(I)$
R_{int} = 0.056
θ_{max} = 31.5°, θ_{min} = 2.2°
$h = -7 \rightarrow 10$
$k = -11 \rightarrow 11$
$l = -53 \rightarrow 49$

Refinement

Refinement on F^2
Least-squares matrix: full
$R[F^2 > 2\sigma(F^2)]$ = 0.039
$wR(F^2)$ = 0.096
S = 1.14
7103 reflections
280 parameters
4 restraints
Primary atom site location: structure-invariant direct methods
Secondary atom site location: difference Fourier map
Hydrogen site location: inferred from neighbouring sites
H atoms treated by a mixture of independent and constrained refinement
$w = 1/[\sigma^2(F_o^2) + (0.042P)^2 + 0.5556P]$ where $P = (F_o^2 + 2F_c^2)/3$
$(\Delta/\sigma)_{max}$ = 0.001
$\Delta\rho_{max}$ = 0.48 e Å^{-3}
$\Delta\rho_{min}$ = −0.26 e Å^{-3}
Absolute structure: 3044 Friedel pairs (Flack, 1983)
Absolute structure parameter: −0.02 (5)

Compound 2 - X-ray

Special details

Refinement

Refinement of F^2 against ALL reflections. The weighted R-factor wR and goodness of fit S are based on F^2, conventional R-factors R are based on F, with F set to zero for negative F^2. The threshold expression of $F^2 > \sigma(F^2)$ is used only for calculating R-factors(gt) *etc*. and is not relevant to the choice of reflections for refinement. R-factors based on F^2 are statistically about twice as large as those based on F, and R- factors based on ALL data will be even larger.

Fractional atomic coordinates and isotropic or equivalent isotropic displacement parameters (Å²)

	x	y	z	U_{iso}*/U_{eq}
S1	0.86390 (5)	0.48436 (4)	0.415688 (10)	0.01886 (7)
O1	1.04249 (15)	0.50897 (17)	0.40065 (3)	0.0280 (2)
O2	0.78023 (19)	0.61620 (14)	0.43649 (3)	0.0281 (3)
O3	0.44314 (16)	0.15217 (14)	0.42285 (3)	0.0234 (2)
O4	0.61230 (19)	0.09476 (15)	0.37338 (3)	0.0283 (3)
O5	−0.38212 (14)	0.82567 (12)	0.26716 (3)	0.0194 (2)
H5O	−0.350 (3)	0.7293 (15)	0.2617 (6)	0.029*
O6	−0.13492 (15)	1.35392 (12)	0.30818 (3)	0.01836 (19)
H6O	−0.193 (3)	1.405 (3)	0.2919 (5)	0.028*
N1	0.73234 (16)	0.44056 (15)	0.38147 (3)	0.0164 (2)
H1N	0.782 (3)	0.403 (3)	0.3625 (6)	0.020*
N2	0.06465 (15)	0.93163 (14)	0.30947 (3)	0.0146 (2)
C1	0.54606 (18)	0.38326 (17)	0.38839 (4)	0.0161 (2)
H1	0.5012	0.4373	0.4114	0.019*
C2	0.42084 (18)	0.43465 (18)	0.35689 (4)	0.0177 (3)
H2A	0.4761	0.4001	0.3333	0.021*
H2B	0.3026	0.3762	0.3594	0.021*
C3	0.38880 (19)	0.62315 (17)	0.35652 (4)	0.0187 (3)
H3A	0.5055	0.6804	0.3513	0.022*
H3B	0.3477	0.6590	0.3812	0.022*
C4	0.24743 (19)	0.67765 (17)	0.32812 (4)	0.0176 (2)
H4A	0.3026	0.6775	0.3033	0.021*
H4B	0.1437	0.5985	0.3281	0.021*
C5	0.18080 (18)	0.85298 (18)	0.33762 (4)	0.0170 (2)
H5A	0.1115	0.8474	0.3609	0.020*
H5B	0.2881	0.9256	0.3418	0.020*
C6	0.54010 (19)	0.19333 (18)	0.39345 (4)	0.0174 (2)
C7	0.4345 (2)	−0.0252 (2)	0.43097 (5)	0.0268 (3)
H7A	0.3506	−0.0799	0.4138	0.040*
H7B	0.3908	−0.0412	0.4561	0.040*
H7C	0.5561	−0.0744	0.4284	0.040*
C8	−0.10305 (17)	0.83432 (17)	0.30299 (4)	0.0147 (2)
H8A	−0.1743	0.8270	0.3260	0.018*
H8B	−0.0709	0.7192	0.2953	0.018*
C9	−0.21775 (18)	0.91789 (16)	0.27326 (4)	0.0144 (2)
H9	−0.1462	0.9225	0.2499	0.017*
C10	−0.26929 (18)	1.09426 (16)	0.28466 (4)	0.0154 (2)
H10A	−0.3494	1.0904	0.3065	0.019*
H10B	−0.3366	1.1500	0.2645	0.019*
C11	−0.09695 (18)	1.19249 (16)	0.29349 (4)	0.0151 (2)
H11	−0.0233	1.2054	0.2706	0.018*
C12	0.01629 (19)	1.10163 (17)	0.32206 (4)	0.0167 (2)
H12A	0.1289	1.1660	0.3270	0.020*
H12B	−0.0534	1.0940	0.3453	0.020*

C13	0.8672 (2)	0.30655 (18)	0.44437 (4)	0.0181 (2)
C14	0.9471 (2)	0.1581 (2)	0.43214 (4)	0.0204 (3)
H14	1.0053	0.1540	0.4089	0.024*
C15	0.9409 (2)	0.0168 (2)	0.45412 (4)	0.0219 (3)
H15	0.9937	−0.0846	0.4456	0.026*
C16	0.8578 (2)	0.0214 (2)	0.48865 (4)	0.0224 (3)
C17	0.7818 (2)	0.1714 (2)	0.50058 (4)	0.0241 (3)
H17	0.7273	0.1768	0.5242	0.029*
C18	0.7842 (2)	0.3135 (2)	0.47857 (4)	0.0224 (3)
H18	0.7296	0.4144	0.4869	0.027*
C19	0.8532 (3)	−0.1325 (2)	0.51243 (5)	0.0294 (3)
H19A	0.9706	−0.1454	0.5249	0.044*
H19B	0.8298	−0.2311	0.4972	0.044*
H19C	0.7561	−0.1212	0.5307	0.044*
O1W	0.30021 (13)	0.99890 (13)	0.25182 (3)	0.01599 (18)
H1W	0.231 (2)	0.971 (3)	0.2685 (4)	0.024*
H2W	0.3952 (19)	0.948 (2)	0.2564 (6)	0.024*

Atomic displacement parameters (Å²)

	U^{11}	U^{22}	U^{33}	U^{12}	U^{13}	U^{23}
S1	0.01633 (14)	0.01847 (15)	0.02177 (15)	−0.00174 (12)	−0.00271 (12)	−0.00077 (12)
O1	0.0176 (5)	0.0347 (6)	0.0318 (6)	−0.0065 (5)	−0.0022 (4)	0.0047 (5)
O2	0.0354 (6)	0.0199 (5)	0.0292 (6)	−0.0003 (5)	−0.0040 (5)	−0.0075 (4)
O3	0.0288 (5)	0.0173 (5)	0.0241 (5)	−0.0010 (4)	0.0068 (4)	0.0032 (4)
O4	0.0366 (7)	0.0182 (5)	0.0301 (6)	0.0033 (5)	0.0091 (5)	0.0008 (4)
O5	0.0117 (4)	0.0123 (4)	0.0342 (6)	0.0002 (4)	−0.0070 (4)	−0.0051 (4)
O6	0.0214 (5)	0.0123 (4)	0.0214 (5)	0.0028 (4)	−0.0032 (4)	−0.0026 (3)
N1	0.0141 (5)	0.0179 (5)	0.0173 (5)	0.0013 (4)	−0.0017 (4)	0.0008 (4)
N2	0.0115 (4)	0.0121 (5)	0.0202 (5)	0.0005 (4)	−0.0035 (4)	0.0005 (4)
C1	0.0140 (5)	0.0145 (6)	0.0198 (6)	0.0014 (4)	−0.0012 (5)	0.0016 (5)
C2	0.0142 (5)	0.0161 (6)	0.0227 (7)	0.0022 (4)	−0.0038 (5)	0.0009 (5)
C3	0.0142 (6)	0.0164 (6)	0.0256 (7)	0.0011 (5)	−0.0064 (5)	0.0039 (5)
C4	0.0154 (6)	0.0162 (6)	0.0212 (6)	0.0027 (5)	−0.0040 (5)	0.0014 (5)
C5	0.0158 (6)	0.0158 (6)	0.0195 (6)	0.0025 (4)	−0.0062 (5)	0.0004 (5)
C6	0.0152 (5)	0.0184 (6)	0.0187 (6)	0.0012 (5)	−0.0032 (5)	0.0021 (5)
C7	0.0331 (8)	0.0180 (7)	0.0294 (8)	−0.0032 (6)	0.0027 (6)	0.0062 (6)
C8	0.0120 (5)	0.0123 (5)	0.0199 (6)	−0.0010 (4)	−0.0027 (4)	0.0005 (4)
C9	0.0109 (5)	0.0125 (5)	0.0197 (6)	0.0004 (4)	−0.0027 (4)	−0.0015 (4)
C10	0.0141 (5)	0.0122 (5)	0.0200 (6)	0.0012 (4)	−0.0051 (5)	−0.0014 (4)
C11	0.0162 (6)	0.0115 (5)	0.0174 (6)	0.0001 (4)	−0.0031 (5)	−0.0010 (4)
C12	0.0166 (6)	0.0130 (6)	0.0205 (6)	0.0018 (4)	−0.0056 (5)	−0.0028 (5)
C13	0.0145 (5)	0.0223 (6)	0.0175 (6)	0.0001 (5)	−0.0032 (5)	−0.0004 (5)
C14	0.0217 (6)	0.0234 (7)	0.0161 (6)	0.0009 (5)	0.0000 (5)	−0.0028 (5)
C15	0.0237 (6)	0.0223 (7)	0.0197 (6)	0.0024 (6)	−0.0027 (5)	−0.0010 (5)
C16	0.0199 (6)	0.0284 (7)	0.0189 (6)	−0.0026 (6)	−0.0050 (5)	0.0032 (5)
C17	0.0192 (6)	0.0358 (8)	0.0173 (6)	0.0009 (6)	0.0004 (5)	0.0000 (6)
C18	0.0176 (6)	0.0289 (7)	0.0206 (7)	0.0045 (6)	−0.0017 (5)	−0.0026 (6)
C19	0.0339 (8)	0.0307 (8)	0.0236 (7)	−0.0056 (7)	−0.0049 (7)	0.0077 (6)
O1W	0.0127 (4)	0.0128 (4)	0.0225 (5)	0.0006 (3)	−0.0013 (3)	0.0017 (4)

Geometric parameters (Å, º)

S1—O1	1.4345 (12)	C7—H7B	0.9800
S1—O2	1.4358 (12)	C7—H7C	0.9800
S1—N1	1.6159 (13)	C8—C9	1.5262 (18)
S1—C13	1.7639 (15)	C8—H8A	0.9900
O3—C6	1.3280 (18)	C8—H8B	0.9900
O3—C7	1.4486 (19)	C9—C10	1.5170 (18)
O4—C6	1.1986 (18)	C9—H9	1.0000
O5—C9	1.4310 (16)	C10—C11	1.5230 (18)
O5—H5O	0.830 (9)	C10—H10A	0.9900
O6—C11	1.4238 (16)	C10—H10B	0.9900
O6—H6O	0.838 (9)	C11—C12	1.5174 (19)
N1—C1	1.4639 (18)	C11—H11	1.0000
N1—H1N	0.84 (2)	C12—H12A	0.9900
N2—C5	1.4748 (17)	C12—H12B	0.9900
N2—C8	1.4749 (16)	C13—C18	1.389 (2)
N2—C12	1.4769 (17)	C13—C14	1.396 (2)
C1—C2	1.5272 (19)	C14—C15	1.385 (2)
C1—C6	1.529 (2)	C14—H14	0.9500
C1—H1	1.0000	C15—C16	1.399 (2)
C2—C3	1.5241 (19)	C15—H15	0.9500
C2—H2A	0.9900	C16—C17	1.391 (2)
C2—H2B	0.9900	C16—C19	1.505 (2)
C3—C4	1.5290 (19)	C17—C18	1.390 (2)
C3—H3A	0.9900	C17—H17	0.9500
C3—H3B	0.9900	C18—H18	0.9500
C4—C5	1.523 (2)	C19—H19A	0.9800
C4—H4A	0.9900	C19—H19B	0.9800
C4—H4B	0.9900	C19—H19C	0.9800
C5—H5A	0.9900	O1W—H1W	0.825 (9)
C5—H5B	0.9900	O1W—H2W	0.823 (9)
C7—H7A	0.9800		
O1—S1—O2	119.49 (8)	N2—C8—H8A	109.6
O1—S1—N1	106.31 (7)	C9—C8—H8A	109.6
O2—S1—N1	108.11 (7)	N2—C8—H8B	109.6
O1—S1—C13	108.92 (7)	C9—C8—H8B	109.6
O2—S1—C13	106.47 (7)	H8A—C8—H8B	108.2
N1—S1—C13	106.94 (6)	O5—C9—C10	108.09 (10)
C6—O3—C7	115.51 (12)	O5—C9—C8	110.50 (11)
C9—O5—H5O	105.9 (16)	C10—C9—C8	110.44 (11)
C11—O6—H6O	106.1 (15)	O5—C9—H9	109.3
C1—N1—S1	119.53 (10)	C10—C9—H9	109.3
C1—N1—H1N	115.9 (15)	C8—C9—H9	109.3
S1—N1—H1N	117.0 (14)	C9—C10—C11	109.23 (11)
C5—N2—C8	111.68 (11)	C9—C10—H10A	109.8
C5—N2—C12	108.32 (11)	C11—C10—H10A	109.8
C8—N2—C12	109.50 (10)	C9—C10—H10B	109.8
N1—C1—C2	110.40 (11)	C11—C10—H10B	109.8
N1—C1—C6	110.97 (11)	H10A—C10—H10B	108.3
C2—C1—C6	109.90 (12)	O6—C11—C12	106.40 (11)
N1—C1—H1	108.5	O6—C11—C10	112.55 (11)
C2—C1—H1	108.5	C12—C11—C10	110.71 (11)
C6—C1—H1	108.5	O6—C11—H11	109.0

C3—C2—C1	111.41 (12)	C12—C11—H11	109.0
C3—C2—H2A	109.3	C10—C11—H11	109.0
C1—C2—H2A	109.3	N2—C12—C11	110.98 (11)
C3—C2—H2B	109.3	N2—C12—H12A	109.4
C1—C2—H2B	109.3	C11—C12—H12A	109.4
H2A—C2—H2B	108.0	N2—C12—H12B	109.4
C2—C3—C4	113.08 (12)	C11—C12—H12B	109.4
C2—C3—H3A	109.0	H12A—C12—H12B	108.0
C4—C3—H3A	109.0	C18—C13—C14	120.31 (14)
C2—C3—H3B	109.0	C18—C13—S1	119.61 (12)
C4—C3—H3B	109.0	C14—C13—S1	120.04 (11)
H3A—C3—H3B	107.8	C15—C14—C13	119.60 (14)
C5—C4—C3	109.02 (12)	C15—C14—H14	120.2
C5—C4—H4A	109.9	C13—C14—H14	120.2
C3—C4—H4A	109.9	C14—C15—C16	120.88 (14)
C5—C4—H4B	109.9	C14—C15—H15	119.6
C3—C4—H4B	109.9	C16—C15—H15	119.6
H4A—C4—H4B	108.3	C17—C16—C15	118.61 (14)
N2—C5—C4	114.78 (11)	C17—C16—C19	120.95 (14)
N2—C5—H5A	108.6	C15—C16—C19	120.43 (15)
C4—C5—H5A	108.6	C18—C17—C16	121.16 (14)
N2—C5—H5B	108.6	C18—C17—H17	119.4
C4—C5—H5B	108.6	C16—C17—H17	119.4
H5A—C5—H5B	107.5	C13—C18—C17	119.43 (15)
O4—C6—O3	124.55 (14)	C13—C18—H18	120.3
O4—C6—C1	124.44 (14)	C17—C18—H18	120.3
O3—C6—C1	111.01 (12)	C16—C19—H19A	109.5
O3—C7—H7A	109.5	C16—C19—H19B	109.5
O3—C7—H7B	109.5	H19A—C19—H19B	109.5
H7A—C7—H7B	109.5	C16—C19—H19C	109.5
O3—C7—H7C	109.5	H19A—C19—H19C	109.5
H7A—C7—H7C	109.5	H19B—C19—H19C	109.5
H7B—C7—H7C	109.5	H1W—O1W—H2W	104 (2)
N2—C8—C9	110.09 (11)		

O1—S1—N1—C1	170.63 (11)	C8—C9—C10—C11	55.75 (14)
O2—S1—N1—C1	−59.92 (12)	C9—C10—C11—O6	−173.73 (11)
C13—S1—N1—C1	54.38 (12)	C9—C10—C11—C12	−54.79 (15)
S1—N1—C1—C2	150.56 (10)	C5—N2—C12—C11	177.57 (11)
S1—N1—C1—C6	−87.34 (14)	C8—N2—C12—C11	−60.42 (14)
N1—C1—C2—C3	−70.33 (15)	O6—C11—C12—N2	−179.69 (11)
C6—C1—C2—C3	166.94 (12)	C10—C11—C12—N2	57.72 (15)
C1—C2—C3—C4	−173.52 (12)	O1—S1—C13—C18	134.67 (12)
C2—C3—C4—C5	162.20 (12)	O2—S1—C13—C18	4.57 (14)
C8—N2—C5—C4	61.09 (15)	N1—S1—C13—C18	−110.83 (12)
C12—N2—C5—C4	−178.24 (12)	O1—S1—C13—C14	−47.64 (14)
C3—C4—C5—N2	171.18 (11)	O2—S1—C13—C14	−177.73 (12)
C7—O3—C6—O4	2.7 (2)	N1—S1—C13—C14	66.86 (13)
C7—O3—C6—C1	−177.78 (13)	C18—C13—C14—C15	0.9 (2)
N1—C1—C6—O4	−48.0 (2)	S1—C13—C14—C15	−176.75 (12)
C2—C1—C6—O4	74.37 (18)	C13—C14—C15—C16	−0.9 (2)
N1—C1—C6—O3	132.49 (13)	C14—C15—C16—C17	−0.2 (2)
C2—C1—C6—O3	−105.12 (14)	C14—C15—C16—C19	−179.50 (15)
C5—N2—C8—C9	−178.99 (11)	C15—C16—C17—C18	1.3 (2)

C12—N2—C8—C9	61.04 (14)	C19—C16—C17—C18	−179.39 (15)
N2—C8—C9—O5	−179.14 (10)	C14—C13—C18—C17	0.2 (2)
N2—C8—C9—C10	−59.60 (14)	S1—C13—C18—C17	177.85 (12)
O5—C9—C10—C11	176.74 (11)	C16—C17—C18—C13	−1.3 (2)

Hydrogen-bond geometry (Å, º)

D—H···*A*	*D*—H	H···*A*	*D*···*A*	*D*—H···*A*
O5—H5*O*···O1*W*^{i}	0.83 (1)	1.94 (1)	2.7667 (14)	174 (2)
O6—H6*O*···O1*W*^{ii}	0.84 (1)	1.93 (1)	2.7564 (14)	169 (2)
N1—H1*N*···O6^{iii}	0.84 (2)	2.11 (2)	2.9272 (16)	165.3 (19)
O1*W*—H1*W*···N2	0.83 (1)	1.95 (1)	2.7740 (16)	174 (2)
O1*W*—H2*W*···O5^{iv}	0.82 (1)	1.95 (1)	2.7686 (13)	179 (2)

Symmetry codes: (i) −*x*, *y*−1/2, −*z*+1/2; (ii) −*x*, *y*+1/2, −*z*+1/2; (iii) *x*+1, *y*−1, *z*; (iv) *x*+1, *y*, *z*.

Biological assays

Kirby-Bauer testing [33] (or agar diffusion testing) was performed to assess the sensitivity of various bacteria to G*-KPV-NH_2 (**12a**), H-K(G*)PV-NH_2 (**12b**) and Ac-KPV-NH_2 (**12c**). The bacteria tested were W3110: *E. coli* K12; WBB06: *E. coli Δ(rfaC-rfaF)* (an *E. coli* strain missing two genes involved in lipopolysaccharide synthesis and sensitive to numerous antibiotics) [34]; *Salmonella enterica* (ATCC14028); *Vibrio cholera* C6706 [35]; and *Staphyloccocus aureus* USA300 JE2 [36]. Ampicillin and water were used as positive and negative controls, respectively. Disks of filter paper were spotted with 5 μL of water or 10 mg/mL solutions of ampicillin and **12a-c**. Each bacterium and compound were tested by placing the disks on a lawn of bacterial cells and incubating overnight at 37 ˚C. Visual inspection of the plates for zones of inhibited cell growth around each disc was used to determine the sensitivity of the bacteria to each compound.

The sensitivity of *Staphyloccocus aureus* USA300 JE2 to commercially sourced Ac-KPV-NH_2 (**12c**) was determined using liquid cultures resuspended in various buffers. An overnight culture of *Staphylococcus aureus* USA300 JE2 was grown in LB medium. The culture (1 mL) was centrifuged, washed in MOPS minimal medium (Teknova Inc., Hollister CA), and resuspended in the same medium. Cells were diluted to approximately 10^7 cells/mL in MOPS medium, MOPS medium + 10 mM $CaCl_2$, MOPS medium + 10% laked horse blood (LHB) (Thermo-Fisher Scientific), or MOPS medium + 10 mM $CaCl_2$ + 10% LHB , each buffer with and without 0.1 mM Ac-KPV-NH_2 (Bachem, Bubendorf, Switzerland). Cells were incubated for 2 hours at 37 °C at which time viability was determined by colony counting of serially diluted cells.

Fig S1 shows the agar plates with the discs for W3110: *E. coli* K12; WBB06: *E. coli Δ(rfaC-rfaF)* [34]; *Salmonella enterica* (ATCC 14028); *Vibrio cholera*, C6706 [35]; and *Staphyloccocus aureus* USA300 [36]. In each case, as expected, the region of the disc spotted with the positive control (ampicillin, designated with a + on the disc) is surrounded by a zone where cell growth was inhibited.

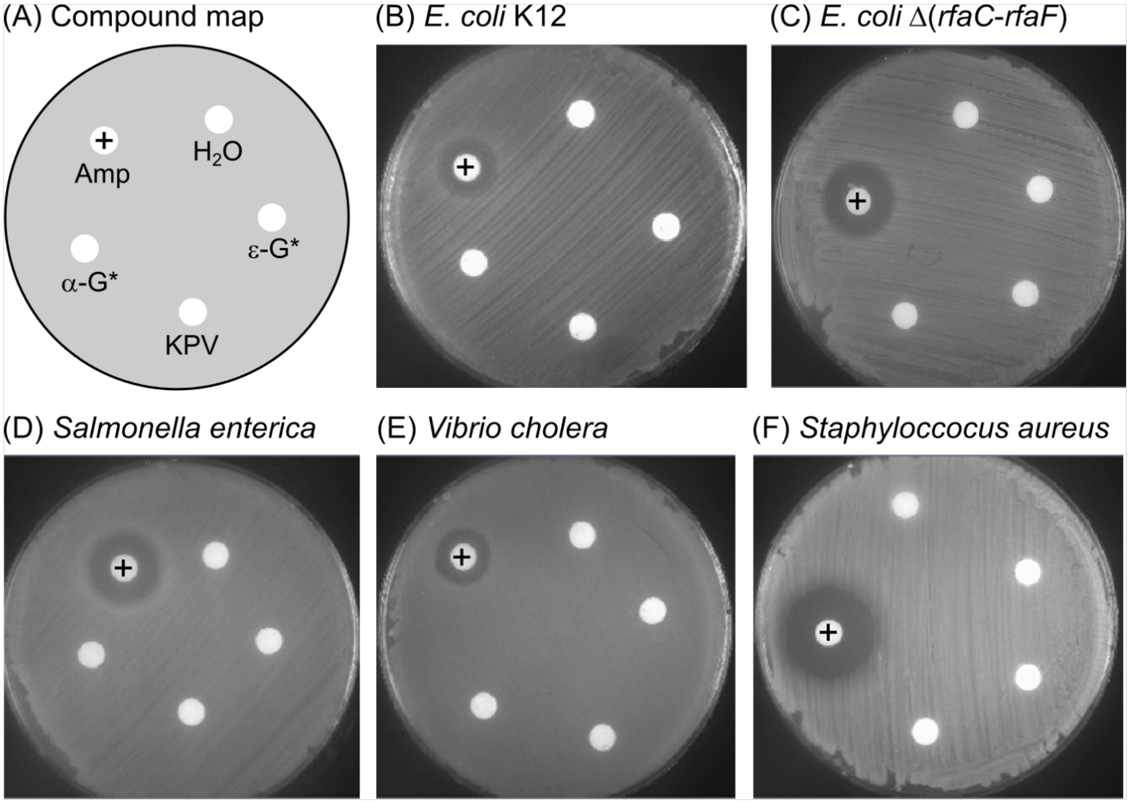

Fig S1. Sensitivity of various bacteria to compounds 12a-c. (a) Compounds, spotted on filter disks and placed on cultures, include ampicillin (designated with +), water, H-K(εG*)PV-NH_2, (**12b**), Ac-KPV-NH_2 (**12c**), and αG*-KPV-NH_2 (**12a**) (starting with ampicillin and moving clock-wise). Several strains of bacteria were tested: (b) W3110: *E. coli* K12; (c) WBB06: *E. coli Δ(rfaC-rfaF)* [34]; (d) *Salmonella enterica* (ATCC 14028; (e) *Vibrio cholera*, C6706 [35]; and (f) *Staphyloccocus aureus* USA300 JE2 [36].

To verify the activity of Ac-KPV-NH_2 (**12c**), the peptide was purchased from Bachem (Bubendorf, Switzerland), the same supplier as was used by Charnley *et al.* [19] Following protocols similar to those reported by Cutuli *et al.* [12] and Charnley *et al.* [19], the commercially sourced Ac-KPV-NH_2 (**12c**) was tested for anti-microbial activity against *Staphylococcus aureus* USA300 JE2. Images of plates used for colony counting are shown in Fig S2. Higher cell-density plates, specifically 100 cells/plate and 1000 cells/plate were also studied (data not shown). Exposure of the cells to the commercial Ac-KPV-NH_2 (**12c**) at 0.1 mM did not affect the viability of the cells. Media with calcium and horse blood were also tested, but these additives had no effect, *i.e.,* there was still no inhibition of bacterial growth (data not shown).

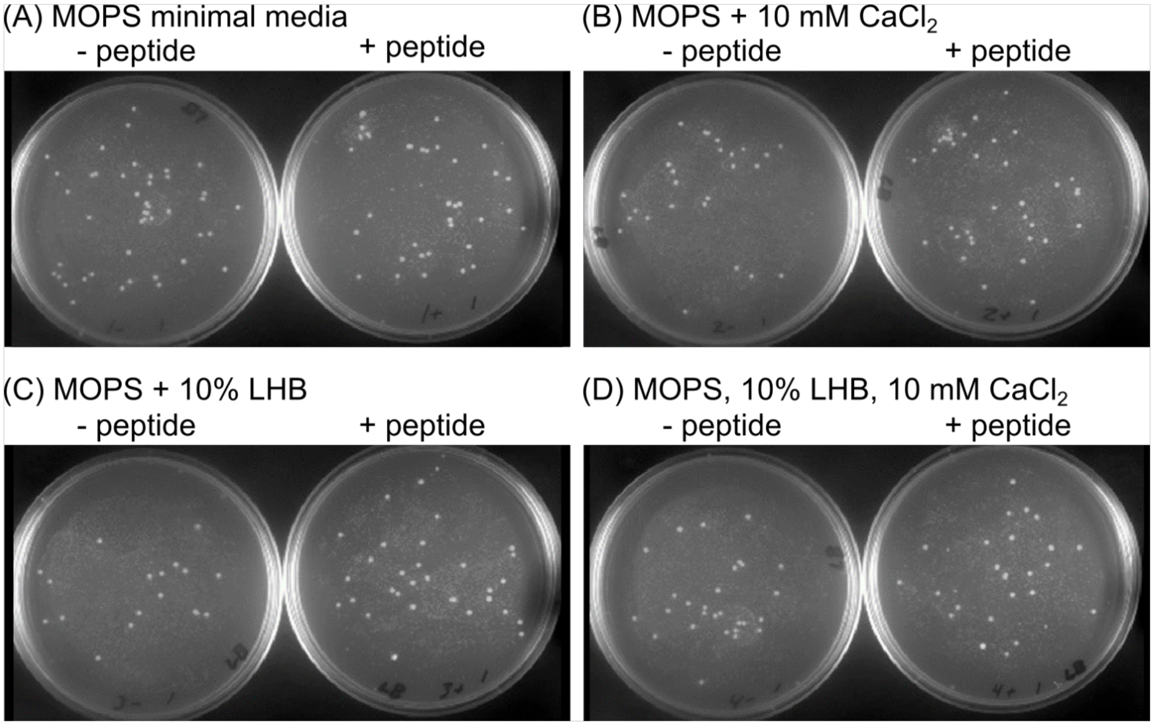

Fig S2. *Staphylococcus aureus* sensitivity to compound 12c in various media. *Staphylococcus aureus* USA300 JE2 cells were incubated in the absence (- peptide) and

presence (+ peptide) of 0.1 mM Ac-KPV-NH_2 (**12c**) in various buffers: (a) MOPS minimal medium, (b) MOPS medium + 10 mM $CaCl_2$, (c) MOPS medium + 10% laked horse blood, and (d) MOPS medium + 10 mM $CaCl_2$ + 10% laked horse blood. Viability was determined by colony counting of serially diluted cells (10 cells/plate).

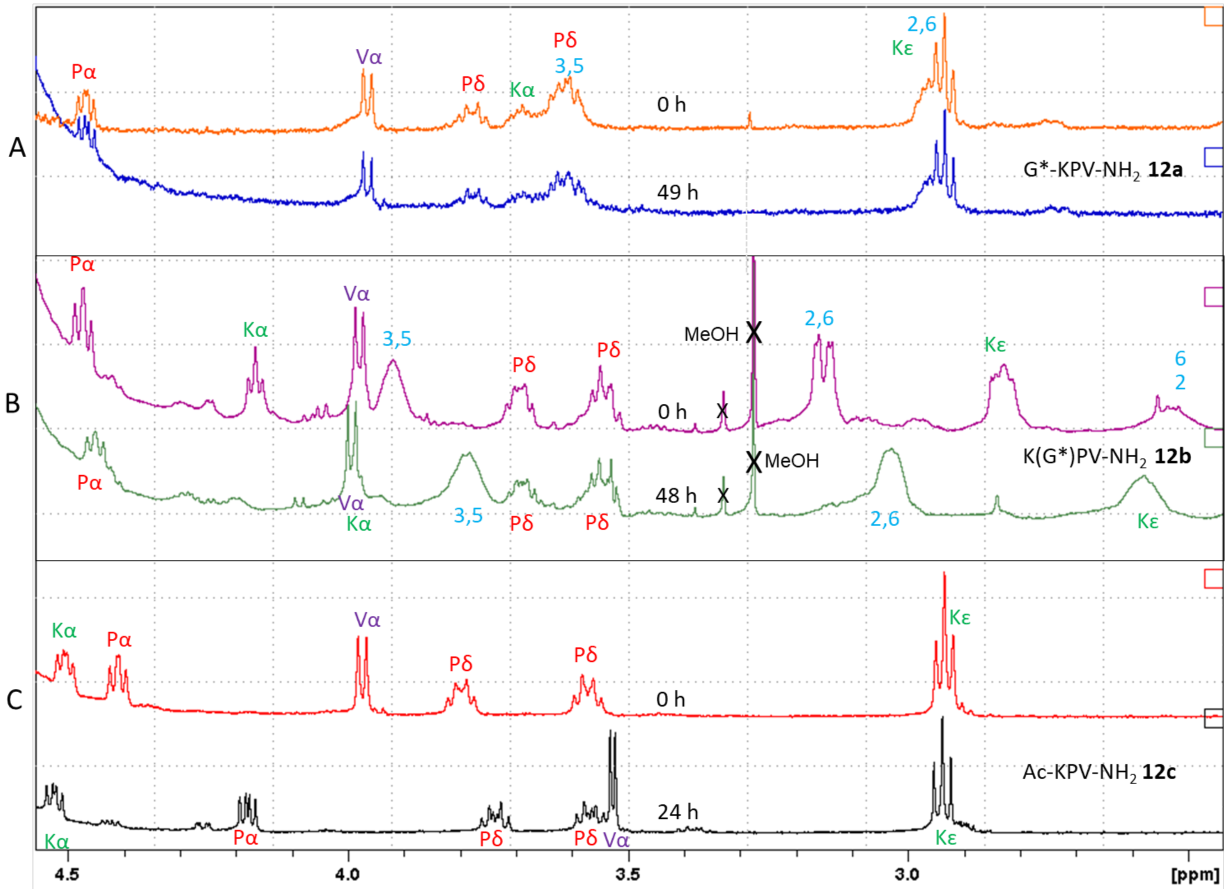

Fig S3. Stability of tripeptides **12a-c** to pronase, monitored by ^{1}H NMR at 500 MHz in D_2O. (A) Compound **12a**; (B) Compound **12b**; and (C) Compound **12c**. Peaks are labeled as follows: Pα = Hα of Pro residue etc. and 3,5 = H3 and H5 of the piperidine moiety.
